# Highly Effective Inhibition of Biofilm Formation by the First Metagenome-Derived AI-2 Quenching Enzyme

**DOI:** 10.3389/fmicb.2016.01098

**Published:** 2016-07-13

**Authors:** Nancy Weiland-Bräuer, Martin J. Kisch, Nicole Pinnow, Andreas Liese, Ruth A. Schmitz

**Affiliations:** ^1^Institute for General Microbiology, Molecular Microbiology, University KielKiel, Germany; ^2^Institute of Technical Biocatalysis, Technical University HamburgHamburg, Germany

**Keywords:** quorum quenching, metagenomic, biofilm inhibition, AI-2, oxidoreductase

## Abstract

Bacterial cell–cell communication (quorum sensing, QS) represents a fundamental process crucial for biofilm formation, pathogenicity, and virulence allowing coordinated, concerted actions of bacteria depending on their cell density. With the widespread appearance of antibiotic-resistance of biofilms, there is an increasing need for novel strategies to control harmful biofilms. One attractive and most likely effective approach is to target bacterial communication systems for novel drug design in biotechnological and medical applications. In this study, metagenomic large-insert libraries were constructed and screened for QS interfering activities (quorum quenching, QQ) using recently established reporter strains. Overall, 142 out of 46,400 metagenomic clones were identified to interfere with acyl-homoserine lactones (AHLs), 13 with autoinducer-2 (AI-2). Five cosmid clones with highest simultaneous interfering activities were further analyzed and the respective open reading frames conferring QQ activities identified. Those showed homologies to bacterial oxidoreductases, proteases, amidases and aminotransferases. Evaluating the ability of the respective purified QQ-proteins to prevent biofilm formation of several model systems demonstrated highest inhibitory effects of QQ-2 using the crystal violet biofilm assay. This was confirmed by heterologous expression of the respective QQ proteins in *Klebsiella oxytoca* M5a1 and monitoring biofilm formation in a continuous flow cell system. Moreover, QQ-2 chemically immobilized to the glass surface of the flow cell effectively inhibited biofilm formation of *K. oxytoca* as well as clinical *K. pneumoniae* isolates derived from patients with urinary tract infections. Indications were obtained by molecular and biochemical characterizations that QQ-2 represents an oxidoreductase most likely reducing the signaling molecules AHL and AI-2 to QS-inactive hydroxy-derivatives. Overall, we propose that the identified novel QQ-2 protein efficiently inhibits AI-2 modulated biofilm formation by modifying the signal molecule; and thus appears particularly attractive for medical and biotechnological applications.

## Introduction

The bacterial cell–cell communication (QS) is based on small signal molecules, so-called autoinducers, and represents a cell density-dependent process effecting gene regulation in Prokaryotes. Intra- and extra-cellular accumulation of autoinducers enables bacteria to detect an increasing cell density and thus allows changing their gene expression to coordinate behaviors that require high cell densities (for review see Dickschat, 2010; Castillo, 2015), e.g., pathogenicity and biofilm formation (Landini et al., 2010; Castillo-Juárez et al., 2015). Among those autoinducers are acyl-homoserine lactones (AHL) in Gram-negative bacteria, short peptide signals in Gram-positive bacteria, and furan molecules known as autoinducer-2 (AI-2) in both groups (Liu et al., 2012; Du et al., 2014; Brackman and Coenye, 2015). In addition, cholera autoinducer I (CAI-1) controlling virulence factor production and biofilm development in *Vibrio cholerae* was identified (Higgins et al., 2007). Recently, AI-3 has been identified as an inter-domain chemical signaling system between microorganisms and their hosts, especially exploited by pathogens like enterohemorrhagic *E. coli* (EHEC) to regulate virulence traits (Moreira and Sperandio, 2010; Kalia, 2015).

QS is known to play a significant role in biofilm formation (Dickschat, 2010; Brackman and Coenye, 2015; Carlier et al., 2015) which can cause material degradation, fouling, contamination, or infections (Elias and Banin, 2012; Mieszkin et al., 2013; Wu et al., 2015). Since biofilm formation is QS dependent, interfering bacterial cell–cell communication is an attractive and novel strategy to prevent and inhibit biofilm formation. Interference with bacterial cell–cell communication (quorum quenching, QQ) can be generally achieved by targeting synthesis, recognition or transport of autoinducers. Moreover, it is also possible to degrade or modify the respective signaling molecules or interfere with the signal perception with antagonistic small molecules. Well-known naturally occurring examples for QQ proteins are (i) AHL-lactonases hydrolyzing the ester bond of the homoserine lactone (HL) ring to inactivate the signaling molecule (Dong et al., 2000; Chen et al., 2013), (ii) AHL-acylases inactivating AHL signals by cleaving its amide bond resulting in the corresponding fatty acids and HL which are not effective as signals (Leadbetter and Greenberg, 2000; Kalia et al., 2011), (iii) AHL-oxidoreductases reducing the 3-oxo group of AHLs to generate corresponding 3-hydroxy derivatives (Uroz et al., 2005; Bijtenhoorn et al., 2011b; Lord et al., 2014). In contrast to various AHL-quenching mechanisms and compounds, only very few AI-2 interfering mechanisms have been reported in detail so far. Those quenching mechanisms are mainly based on interference with AI-2 synthesis by S-ribosyl-homocysteine and transition state analogs (Shen et al., 2006; Singh et al., 2006; Widmer et al., 2007), or antagonistic small molecules as shown in *V. harveyi* and *E. coli* (Ganin et al., 2009; Lowery et al., 2009; Vikram et al., 2011; Roy et al., 2013; Yadav et al., 2014).

In recent years, the majority of investigations aiming to identify novel quorum quenching (QQ) compounds were performed with chemical substance libraries and extracts of pure cultures of bacterial isolates or eukaryotic organisms containing secondary metabolites (Fetzner, 2015; Kalia et al., 2015). Besides, cultivation-independent metagenomic approaches harbor a huge potential to identify novel quorum quenching compounds and mechanisms. Metagenomic approaches generally provide insights in the genetic potential present within a microbial community of a habitat (Handelsman, 2004) and thus, enable to identify novel biotechnologically relevant molecules (Schmeisser et al., 2007; Simon et al., 2009; Piel, 2011; Craig, 2012). However, so far only a limited number of metagenomic screens have been performed to identify novel QQ mechanisms, and only a few approaches demonstrated the ability of those QQ molecules to inhibit biofilm formation (Williamson et al., 2005; Guan et al., 2007; Riaz et al., 2008; Bijtenhoorn et al., 2011a,b; Kisch et al., 2014). Nevertheless, naturally occurring QQ biomolecules have been used in particular as novel therapeutic agents combating resistant microorganisms (Dong et al., 2001; Hentzer et al., 2003; Zhang, 2003; Zhang and Dong, 2004; Kalia and Purohit, 2011). Thus, the goal of this study was to identify novel metagenomic-derived non-toxic biomolecules interfering with AI-2 and AHL based QS processes. Identified QQ proteins were further evaluated concerning their capability to prevent QS modulated biofilm formation, particularly regarding their potential as novel biotechnologically relevant anti-pathogenic compounds.

## Materials and methods

### Bacterial strains and plasmids

Bacterial strains used are listed in Table 1. Plasmid DNA was transformed into *E. coli* and *K. oxytoca* cells as previously described (Inoue et al., 1990).

**Table 1:** **Bacterial strains and plasmids used in this study**.

**Strain**	**Description**	**References**
*E. coli* DH5α	F-ø80d*lac*ZΔM15 *rec*A1 Δ(*lac*ZYA*arg*F) U169*deo*R *end*A1 *hsd*R17(rk^−^mk^+^) *pho*A *sup*E44 λ- *thi*-1 *gyr*A96 *rel*A1	Hanahan, 1983
*E. coli* EPI100™-T1^R^	F^−^mcrA Δ(*mrr*-*hsd*RMS-*mcr*BC) ø80d*lac*ZΔM15 Δ*lac*X74 *rec*A1*end*A1 *ara*D139 Δ(*ara, leu*)7697 *gal*U *gal*K λ–*rps*L *nup*G	Epicenter, Madison, USA
*E. coli* EPI300™-T1^R^	F- mcrA Δ(*mrr-hsd*RMS-*mcr*BC) ø80d*lac*Z ΔM15 Δ*lac*X74 *rec*A1 *end*A1 *ara*D139 Δ(*ara,leu*)7697 *gal*U *gal*Kλ- *rps*L *nup*G *trf*A *ton*A *dhfr*	Epicenter, Madison USA
*E. coli* BL21 (DE3)	F^−^ ompT gal dcm lon hsdSB(${\text{r}}_{\text{B}}^{-}$ ${\text{m}}_{\text{B}}^{-}$) λ(DE3 [lacI lacUV5-T7 gene 1 ind1 sam7 nin5])	Studier and Moffatt, 1986
XL1-Blue	*endA1 gyrA96(nalR) thi-1 recA1 relA1 lac glnV44* F'[::Tn*10 proAB lacIq* Δ*(lacZ)*M15] *hsdR17*(${\text{r}}_{\text{K}}^{-}$ ${\text{m}}_{\text{K}}^{-}$)	Stratagene, La Jolla, CA
AI1-QQ.1	reporter strain to identify AHL-QQ compounds	Weiland-Bräuer et al., 2015
AI2-QQ.1	reporter strain to identify AI-2-QQ compounds	
XL1-Blue/pZErO-2	control strain	
*Klebsiella oxytoca* M5a1 wildtype	DSM 7342	DSMZ
*Klebsiella pneumoniae* clinical isolate	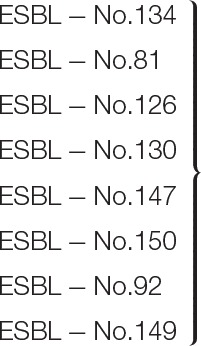	Prof. Dr. Podschun, (National Reference Laboratory for *Klebsiella* species, Kiel University)
*Klebsiella pneumoniae* clinical isolate		
*Klebsiella pneumoniae* clinical isolate		
*Klebsiella pneumoniae* clinical isolate		
*Klebsiella pneumoniae* clinical isolate		
*Klebsiella pneumoniae* clinical isolate		
*Klebsiella pneumoniae* clinical isolate		
*Klebsiella oxytoca* clinical isolate		
*Bacillus subtilis*	DSM 6887	DSMZ
*Staphylococcus aureus*	DSM 11823	DSMZ
*Pseudomonas aeruginosa* PAO1	DSM 1707	DSMZ
**Plasmid**	**Description**	**References**
pCC1FOS™ pWEB-TNC™	Fosmid Cosmid	Epicenter, Madison, USA
pCR®II-TOPO®	TA-cloning vector	Invitrogen, Karlsruhe, Germany
pDrive	Cloning vector	Qiagen, Hilden, Germany
pMAL-c2X	Cloning vector encoding maltose binding protein	NEB, Frankfurt, Germany
pZERrO-2	Cloning vector, *ccdB* under transcriptional control of the *lac* promoter	Life Technologies, Darmstadt, Germany
pRS488	*ccdB* under control of the *luxI* promoter	Weiland-Bräuer et al., 2015
pRS489	*ccdB* under control of the *lsrA* promoter	
pRS611	QQ-2 in pMAL-c2X	This study
pRS612	QQ-3 pMAL-c2X	This study
pRS613	QQ-4 in pMAL-c2X	This study
pRS614	QQ-5 in pMAL-c2X	This study
pRS615	QQ-6 in pMAL-c2X	This study
pRS616	QQ-7 in pMAL-c2X	This study
pRS617	QQ-8 in pMAL-c2X	This study
pRS618	QQ-9 in pMAL-c2X	This study
pRS619	QQ-10 in pMAL-c2X	This study
pRS620	QQ-11 in pMAL-c2X	This study
pRS621	QQ-12 in pMAL-c2X	This study

### Growth media

Media used were Luria-Bertani (LB) medium (Sambrook et al., 1989), GC minimal medium (with 1% (v/v) glycerol as carbon and energy source and 0.3% (w/v) casamino acids) (Gerlach et al., 1988), Caso Bouillon (17 g/L Casein peptone, 3 g/L soybean peptone, 5 g/L NaCl, 2.5 g/L K_2_HPO_4_, 2.5 g/L glucose) and AB minimal medium (200 mL solution A: 15 mM (NH_4_)_2_SO_4_, 42 mM Na_2_HPO_4_, 22 mM KH_2_PO_4_, 51 mM NaCl; combined with 800 mL solution B: 0.1 mM CaCl_2_, 1 mM MgCl_2_, 3 μM FeCl_3_; supplemented with 0.4 % (w/v) glucose). When indicated, the medium was supplemented with final concentrations of the following antibiotics ampicillin (100 μg/mL), kanamycin (30 μg/mL) or chloramphenicol (12.5 μg/mL).

### Sampling for DNA extraction

#### Water sampling

Surface water was collected near Stein, Baltic Sea, Germany (54.25°N, 10.16°E) in 5 m depth in May 2008 using a membrane pump on board of the ship Polarfuchs (Helmholtz Centre, Kiel). Collected samples from the potentially high productive surface layer were pre-filtrated with filters of 10 μm pore size, directly followed by a consecutive filtration of 2 L with polycarbonate membrane filters of 0.22 μm pore size. Surface water samples (2 L) taken from a reservoir of a flooded salt marsh, Hamburger Hallig, Germany (54.36°N, 8.49°E) in September 2005 were pre-filtrated with filters of 50 μM and 10 μM pore size, followed by a filtration of 500 mL with polycarbonate membrane filters of 0.22 μm pore size.

#### Sampling from *Aurelia aurita*

*A. aurita* was sampled in August 2006 (54.28°N, 9.50°E) and July 2008 (54.22°N, 10.23°E) in the Baltic Sea near Kiel, Germany. Medusae were thoroughly rinsed three times with sterile seawater to remove loosely attached microorganisms and an area of approx. 5 cm^2^ was swabbed with a sterile cotton-tipped applicator (Weiland et al., 2010).

#### Sampling of biofilm

Biofilm from a washing machine (household in North Germany (3 persons) washing machine, detergent dispenser) was removed with sterile instruments from the dispensing compartment. Samples from Black Sea were obtained from cruise 317-2 of research vessel (RV) “Poseidon” to the lower Crimean shelf of the Northwest Black Sea in September 2004. By using the manned submersible “Jago,” a sample of a microbial mat associated with a carbonate column was taken at water depth of approximately 230 m (44.46°N, 31.59°E). The samples were immediately frozen on board and stored at −20°C.

#### Sampling of cryoconite

The field study was performed in September 2006 on Jamtalferner glacier (47.51°N, 10.09°E), Austria. The cryoconite sample was collected near the glacier base at 2700 m above sea level using a sterile 500 mL bottle and immediately transferred to the lab.

### DNA isolation procedures

Metagenomic DNA was extracted by direct lysis according to a modified protocol of Henne et al. (1999) described in detail in Weiland et al. (2010). Cosmid/fosmid DNA was isolated from 5 mL overnight cultures of metagenomic clones using High-Speed-Plasmid-Mini Kit (Avegene, Taiwan).

### Construction of metagenomic large-insert libraries and preparation of cell extracts and culture supernatants

Large-insert cosmid libraries were constructed using pWEB-TNC™ Cosmid Cloning Kit (Epicenter, Madison/USA) according to the protocol of the manufacturer; fosmid libraries were constructed using Copy Control™ Fosmid Library Production Kit with vector pCC1FOS (Epicenter, Madison/USA) with modifications (see Weiland et al., 2010). Metagenomic clones were grown in 200 μL LB medium and stored in 96 well plates at −80°C supplemented with 8% (v/v) DMSO. The following libraries were constructed: III, Black Sea; IV, water column salt marsh; X, *A. aurita* surface sampled in 2006; XIII, cryoconite; XIV, biofilm of a washing machine; XVII, water column Baltic Sea near Stein; XIX, *A. aurita* surface sampled in 2008, ranging from 3000 to 14,800 metagenomics clones per library (see Table S1). Preparation of cell-free culture supernatants and cell extracts of pools of 96 metagenomic clones and individual clones of QQ positive 96er pools was performed as described in Weiland-Bräuer et al. (2015).

### Quorum quenching assay

QQ assays on plates using strains AI1-QQ.1 and AI2-QQ.1 containing the *ccdB* gene under an autoinducer-inducible promoter were performed with cell-free supernatants and cell extracts of metagenomic clones and purified proteins as previously described in Weiland-Bräuer et al. (2015).

### Molecular analysis of quorum sensing interfering cosmid clones

In order to identify the respective ORFs of the cosmids conferring QQ activity a combination of two alternative methods, subcloning and *in vitro* transposon mutagenesis, were used as previously described in Weiland-Bräuer et al. (2015).

### Expression and purification of QQ proteins as maltose binding protein (MBP)-fusions

Putative QQ-ORFs were PCR-cloned into pMAL-c2X N-terminally fusing the QQ ORFs to the maltose binding protein (MPB) using ORF-specific primers adding restriction recognition sites flanking the ORFs (pRS611 – pRS622) (see Table S2); overexpressed and purified as recently described in Weiland-Bräuer et al. (2015).

### Control assay to exclude effects of QQ proteins on the toxicity of CcdB

Additional control experiments were performed to exclude the possibility that QQ proteins affect the toxicity of the lethal protein CcdB (e.g., by degradation or transportation out of the cell). Control plates were prepared with LB agar containing 0.8% agar at 50°C supplemented with final concentrations of 10 mM IPTG, 30 μg/mL kanamycin, and 10 % (vol/vol) exponentially growing culture of XL1-Blue/pZErO-2 containing the *ccdB* gene under control of the *lac* promoter. 5 μL of purified MBP and MBP-QQ fusion proteins were applied on topagar (0.1 μg, 1 μg and 10 μg) and incubated at 37°C.

### Biofilm formation assays

#### Inhibition of biofilm formation using model organisms

*E. coli* K12 MG1655, *Pseudomonas aeruginosa, Bacillus subtilis*, and *Staphylococcus aureus* were grown in 96-well plates in minimal medium (*B. subtilis* and *E. coli*, AB medium; *S. aureus* and *P. aeruginosa*, Caso bouillon) for 24 h at 80 rpm and 37°C, except *B. subtilis*, which was grown at 30°C. Purified MBP-QQ proteins (10, 50, and 100 μg) were added to freshly inoculated cultures (150 μL) in MTPs. After 24 h biofilm formation was monitored and quantified using the crystal violet assay and measuring the absorbance at 590 nm as described by Mack et al. and Djordjevic et al. (Mack and Blain-Nelson, 1995; Djordjevic et al., 2002).

#### Monitoring biofilm formation of *K. oxytoca* M5a1 in continuous flow-cells

Formation of biofilms was monitored using two-channel flow cells constructed of V10A stainless steel. The individual channel dimensions were 3 × 8 × 54 mm (total volume of 1.3 mL). Standard borosilicate glass cover slips (24 × 60 mm; thickness, 0.17 mm) were fixed on the upper and lower side of the flow cell using additive-free silicone glue. Tygon tubes (inner diameter 3.17 mm) were used for connecting the flow cells with a 16-channel Ismatec IPC-N peristaltic pump (Ismatec, Wertheim-Mondfeld, Germany) to connect four two-channel flow cells in parallel. Prior to inoculation, the flow chamber was rinsed with sterile GC minimal medium for 5 h at a flow rate of 20 mL h^−1^. 1.3 × 10^8^ cells / mL of the respective *Klebsiella* strains were added to the chamber and medium flow was arrested for 1 h allowing adhesion of bacterial cells. Flow cells were run at 30°C for 72 h at a rate of 20 mL/h using GC medium supplemented with 30 μM IPTG. After 72 h, biofilms were stained with Live/Dead viability Kit (Invitrogen, Karlsruhe, Germany) according to the instructions of the manufacturer. The entire three-dimensional biofilm structure was recorded by scanning along the biofilm depth using TCS SP confocal laser scanning microscope (Leica, Wetzlar, Germany) and recording the stacks of cross sections simultaneously at corresponding excitation wavelengths of 488 nm (Syto9) and 536 nm (propidium iodide). For each flow cell channel, five image stacks were acquired. For image analysis, three independent biological replicates with two technical replicates were quantified. For each field of view, an appropriate number of optical slices were acquired with a Z-step of 1 μm. Digital image acquisition, post-processing, analysis of the CLSM optical thin sections, three-dimensional reconstructions and calculation of biofilm characteristics were performed with the corresponding Leica software (provided for the TCS SP confocal laser scanning microscope). Statistical analyses were performed with GraphPad Prism 6 software (GraphPad, San Diego, CA, USA). Unpaired *t*-tests were used to compare biofilm characteristics thickness and volume. *P*-values < 0.02 were considered as significant. The respective *P*-values are given in **Tables 3**, **4**.

### Covalent immobilization of QQ-2 on glass surfaces

Borosilicate glass slides (Roth, Karlsruhe, Germany) were coated by the company Surflay Nanotec (Berlin, Germany) with ethyleneimine polymers (PEI) according to the previously published Layer-by-Layer method (Peyratout and Dähne, 2004). Glutaraldehyde (5 % v/v) was incubated on the glass slides for 1 h at 4°C for binding to the amino groups of the PEI. The glass slides were washed three times with water and once with 0.1 M phosphate buffered saline (PBS, pH 7.0). Protein solutions with concentrations between 0.083 and 83.3 μg/mL of the respective QQ protein in PBS were incubated on the slides overnight at 4°C to covalently immobilize the enzymes to the glutaraldehyde. The slides were washed three times with 0.1 M PBS and stored at 4°C for maximal 24 h without losing activity.

### Oxidoreductase assay

1 mM N-(ß-ketocaproyl)-L-homoserinelactone or 1 mM 4-hydroxy-5-methyl-3-furanone were incubated with 0.1 mg purified protein MBP or MBP-QQ-2 in a total reaction volume of 200 μL in 1x PBS pH 8.0 at room temperature (RT). A potential oxidoreductase activity of QQ-2 was assayed by following the decrease of 340 nm absorbance after starting the reaction with 1 mM NADH using a Spectra Max Plus 384 plate reader up to 180 min (Molecular Devices, Biberach, Germany).

### Random mutagenesis of QQ-2 by PCR amplification

QQ-2 ORF was PCR amplified using pRS611 as template, primer set QQ-2_for_ (5′-AATGCTTATGATATTTGAAAA-3′) and QQ-2_rev_ (5′-TTACCGCGGCGCCATA-3′), and *Taq*-DNA polymerase (Thermo Fisher Scientific, Darmstadt, Germany) under conditions of reduced *Taq* polymerase accuracy in the presence of 160 μM MnCl_2_ (Cadwell and Joyce, 1992). The resulting mutated PCR products were purified and TA-cloned into pCRII-TOPO (Life Technologies, Darmstadt, Germany). Mutated QQ-2 ORFs were subsequently excised using the additional restriction sites and cloned into the respective digested pRS611. Resulting clones were analyzed to detect loss of QQ activity (see above) caused by random mutagenesis. The respective plasmid insert of 188 clones, which showed no detectable QQ activity, were sequenced using the primer set QQ-2_for_ and QQ-2_rev_ to determine the mutation rate, identifying nucleotide changes and the respective amino acid (aa) changes and their effects on the predicted protein structure. The aa sequences obtained from mutated non-functional QQ-2 derivatives were compared with homologous sequences using STRAP—Interactive Structure based Sequences Alignment Program (http://www.bioinformatics.org/strap/), followed by prediction of secondary structures, active sites and strictly conserved residues (≥90% conservation).

### Determination of AHL degradation by HPLC/MS/MS analytics

An AHL degradation assay was performed using N-(ß-ketocaproyl)-homoserine lactone (3-oxo-C6-AHL; Sigma-Aldrich, Munich, Germany) as substrate. 50 μM N-(ß-ketocaproyl)-L-homoserine lactone and no (blind control) or 1 mg/mL of the respective enzyme (MBP, control; QQ-2, quenching protein) were incubated in 0.1 M PBS (pH 7.0) in a total reaction volume of 500 μL at 30°C for 14 h. 100 μL fractions were taken from the reaction mixture, 300 μL ethyl acetate (acidified with formic acid for AHS measurements) were added to the sample, and phases were allowed to separate. 100 μL of the organic phase were evaporated at RT and 100 mbar and redissolved in 100 μL acetonitrile. HPLC/MS/MS analysis of the reactant (3-oxo-C6-HSL) and possible degradation products (3-oxo-C6-homoserine, 3-hydroxy-C6-homoserine lactone, 3-hydroxy-C6-homoserine) were carried out by the Central Laboratory of Analytical Chemistry at Hamburg University of Technology using an Agilent 1200 HPLC system with Agilent 1200 autosampler, Agilent 1200 binary pump, Agilent 1260 column oven equipped with a Synergi™ Fusion-RP 150/3 mm, 4 μm, 100 A from Phenomenex (Torrance, CA, USA) and an API 2000 triple quadrupole detector from ABSciex (Framingham, MA, USA). For acyl-homoserine lactone (AHL) measurements, a solvent gradient program (Table S3) was used with double distilled (dd) H_2_O and acetonitrile (ACN) containing each 1% acetic acid as solvents A and B. The following calibration curve was generated by adjusting 0 – 50 μM (steps of 5 μM) 3-oxo-C6-HSL in 0.1 M PBS (pH 7.0) in a total volume of 100 μL: *f(x)* = 3.21 × 10^5^ + 2.46 × 10^5^ ×, *R*^2^ = 0.995. Measurements were performed with three biological and each three technical replicates. Standard deviation for single AHL measurements is 4.5%. Acyl-homoserine (AHS) was also measured using HPLC/MS/MS, but no calibration curves were generated due to the lack of commercially available standards. For AHS measurements, the solvent program was adjusted (see also Table S3). Mass spectrometry settings were as follows: scan type: MRM, polarity: positive, ion source: turbo spray, curtain gas: 20, collision gas (nitrogen): 3, ion spray voltage: 5500 V. Quadrupole settings for AHL and AHS analytics with a dwell time of 50 ms and the following abbreviations: mass settings quadrupole 1 (Q1), mass settings quadrupole 3 (Q3), declustering potential (DP), focusing potential (FP), entrance potential (EP), collision cell entrance potential (CEP) collision energy (CE) and collision cell exit potentials (CXP) (see Table S4). Homoserine lactone masses were measured together with the released acyl chain. Mass settings of the mass detector differ from the exact mass due to hydrogen protonation or deprotonation.

### Nucleotide sequence accession numbers

Sequences of QQ-ORFs QQ-2 to QQ-12 were submitted to GenBank (Asseccion No. JX870904 - JX870914).

## Results

### Characterization of QS-interfering metagenomic clones

Aiming to identify novel metagenomic derived QS interfering biomolecules seven metagenomic large-insert libraries were constructed in *E. coli* from various habitats including microbial consortia which are naturally associated to biological or non-living surfaces (see Table S1). These libraries comprise altogether 46,400 metagenomic clones with approx. 1280 Mbps of metagenomic information. The libraries were analyzed regarding QQ activities using our recently established reporter systems (Weiland-Bräuer et al., 2015) resulting in a total of 87 individual clones conferring AHL-QQ activity (63 cell extracts, 79 supernatants; for details see Table S1). AI-2 interference was detected in 13 individual clones (3 cell extracts, 10 supernatants, see Table S1). Four clones conferring pronounced and simultaneous interference with both signaling molecules were selected for further analysis (III 6/G5, IV 5/E10, IV 5/G8, IV 13/B4), as well as one clone showing exclusively AI-2 interference (IV 5/G7) (see Table 2). The identification of the respective open reading frames (ORFs) conferring QQ activity was achieved by subcloning or transposon mutagenesis followed by sequence analysis.

**Table 2:** **Characterization of identified metagenomic ORFs conferring QQ activities**.

**Original clone designation**	**Characterization of identified potential QQ-ORF**	**Plasmid designa-tion**	**QQ activity of purified MalE-fusion protein**
Black Sea III 6/G5	QQ-11: 309 aa	pRS620	n. d.
	- Closest homolog:		
	. AC: WP_041974651 (56 % aa identity)		
	. radical SAM protein from *Geobacter sp.* OR-1 (308 aa)		
	QQ-12: 478 aa	pRS621	AHL + AI-2
	- Closest homolog:		
	. AC: WP_034270149 (54 % aa identity)		
	. aminotransferase from *Actinospica robinae* (460 aa)		
Salt Marsh, Hamburger Hallig, Germany IV 5/G8	QQ-2: 257 aa	pRS611	AHL + AI-2
	- Closest homolog:		
	. AC: WP_044050964 (99 % aa identity)		
	. 3-hydroxy-2-methylbutyryl-CoA dehydrogenase from *Planktomarina temperata* (255 aa)		
IV 5/G7	QQ-3: 177 aa	pRS612	AI-2
	- Closest homolog:		
	. AC: ADD95869 (32 % aa identity)		
	. hypothetical protein from uncultured organism (336 aa)		
IV 5/E10	QQ-4: 444 aa	pRS613	AHL + AI-2
	- Closest homolog:		
	. AC: WP_052225045 (42 % aa identity)		
	. hypothetical protein from *Mesorhizobium sp.* F7 (518 aa) belonging to Ferredoxin reductase superfamily		
IV 13/B4	QQ-5: 373 aa	pRS614	AHL + AI-2
	- Closest homolog:		
	. AC: WP_048599102 (99 % aa identity)		
	. 4-hydroxy-3-methylbut-2-en-1-yl diphosphate synthase from *Nereida ignava* (373 aa)		
	QQ-6: 373 aa	pRS615	AHL + AI-2
	- Equal to QQ-5 but with 4 random point mutations		
	QQ-7: 217 aa	pRS616	AHL + AI-2
	- Closest homolog:		
	. AC: WP_048599137 (100 % aa identity)		
	. 3-beta hydroxysteroid dehydrogenase from *N. ignava* (273 aa)		
	QQ-8: 376 aa	pRS617	AHL
	- Closest homolog:		
	. AC: WP_048599109 (99 % aa identity)		
	. DNA-binding protein from *N. ignava* (801 aa) containing Lon protease domain		
	QQ-9: 424 aa	pRS618	AHL + AI-2
	- Closest homolog:		
	. AC: WP_048599099 (100 % aa identity)		
	. hypothetical protein from *N. ignava* (424 aa) belonging to N-acetylmuramoyl-L-alanine amidase superfamily		
	QQ-10: 406 aa	pRS619	n. d.
	- Closest homolog:		
	. AC: WP_048599133 (100 % aa identity)		
	. 1-aminocyclopropane-1-carboxylate deaminase from *N. ignava* (392 aa)		

After expression and purification as MBP-fusion proteins selected QQ-ORFs were analyzed using reporter strains AI1-QQ.1 and AI2-QQ.2 (Weiland-Bräuer et al., 2015). AC, Accession number; aa, amino acids; n. d., not detected.

Amino acid sequence analysis revealed that most of the QQ-ORFs show similarity to bacterial oxidoreductases (QQ-2, QQ-4, QQ-5, QQ-6, and QQ-7), proteases (QQ-8), amidases (QQ-9) and aminotransferases (QQ-12) (see Table 2). To confirm the predicted QS interfering activities, the identified ORFs were expressed in *E. coli*, N-terminally fused to maltose binding protein (MBP) and purified by affinity chromatography. Purified fusion proteins were evaluated regarding their quenching activity using the reporter systems (Weiland-Bräuer et al., 2015). The assay demonstrated simultaneous quenching activities against AHL and AI-2 in case of QQ-2, QQ-4, QQ-5, QQ-6, QQ-7, QQ-9, and QQ-12; in contrast, QQ-8 showed exclusive AHL-, and QQ-3 exclusive AI-2 quenching activity (see Table 2). Predicted quenching proteins QQ-10 and QQ-11 showed no QQ activities and were therefore not characterized further. To confirm the presence of QQ activities and exclude that the detected effects of the QQ proteins are based on direct effects on the toxicity of the lethal protein CcdB in the reporter system, an additional control experiment was performed. The plasmid pZErO-2 containing the lethal *ccdB* gene under control of the *lac* promoter was used. In the presence of 10 mM IPTG, the respective XL1-Blue strain carrying pZErO-2 was not able to grow in topagar. The application of QQ proteins did not result in re-establishment of growth (see Figure S1), strongly arguing that the active QQ proteins did not affect the toxicity of CcdB.

### Evaluating effects of identified QQ proteins on biofilm formation *in vitro* and *in vivo*

**Figure 1:**
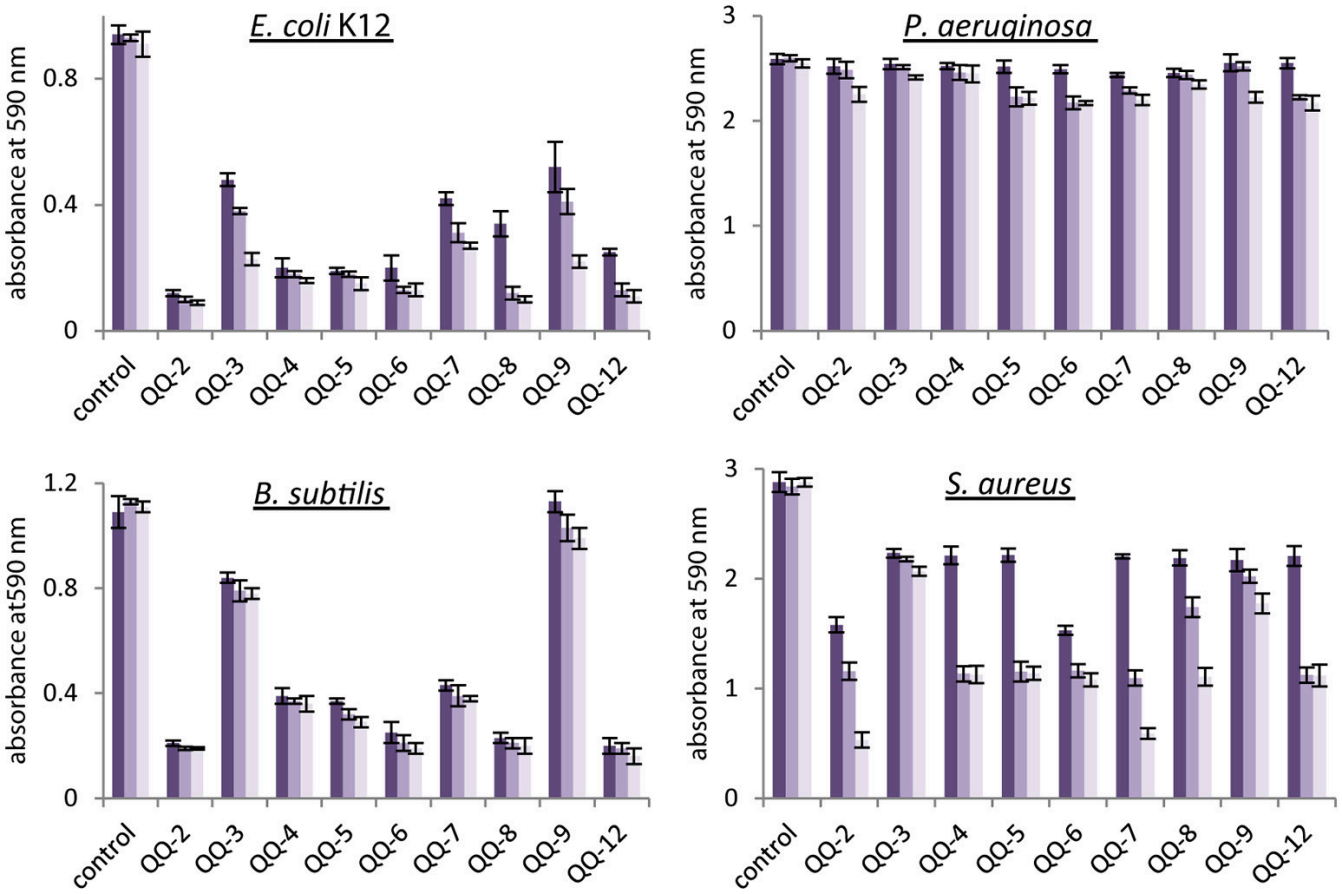
**Inhibition of biofilm formation by identified MBP-QQ proteins**. Biofilm-forming strains (*E. coli, P. aeruginosa, B. subtilis*, and *S. aureus*) were grown in 96 well plates in minimal medium (see Materials and Methods). Purified MBP-QQ proteins were added to 150 μL cultures prior to incubation in amounts of 10 μg (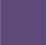), 50 μg (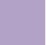), and 100 μg (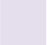). After 24 h, the established biofilms was quantified by crystalviolet (see Materials and Methods). Diagrams represent the average of three independent experiments each performed with three technical replicates.

The influence of the MBP-QQ proteins on one of the QS-dependent processes, i.e., biofilm formation, was studied using four biofilm-forming model organisms, including bacteria of medical and biotechnological interest, *E. coli* K12, *P. aeruginosa, B. subtilis*, and *S. aureus*. In contrast to most Gram-negative bacteria, *E. coli* does not synthesize AHLs (Van Houdt et al., 2006). Here, the formation of micro-colonies and biofilms is dependent on AI-2 (González Barrios et al., 2006; Beloin et al., 2008). The opportunistic pathogenic Gram-negative bacterium *P. aeruginosa* coordinates formation of biofilms by AHL-dependent QS (3-oxo-C12-HSL and C4-HSL) (Davies et al., 1998; Sauer et al., 2002). For the Gram-positive bacteria *B. subtilis* and *S. aureus*, both oligopeptides and AI-2 are essential as signaling molecules to induce biofilm formation (Ren et al., 2002; Yarwood et al., 2004; Lombardía et al., 2006). However, the extents to which the different signaling molecules are involved in the formation of biofilms have not been elucidated yet. Increasing amounts of purified MBP-fusion proteins with verified QQ activity (10, 50, and 100 μg) were added to 150 μL freshly inoculated cultures in microtiter plates. After 24 h incubation under static conditions, biofilm formation was evaluated using the crystal violet biofilm assay (see Materials and Methods). AI-2 modulated biofilm formation of *E. coli* was in general efficiently inhibited even in the presence of small amounts of QQ proteins (see Figure 1). In contrast, the compact biofilms of *P. aeruginosa* were practically not affected. Biofilm formation of *B. subtilis* was almost completely inhibited by QQ-2, QQ-6, QQ-8 and QQ-12, whereas biofilm formation of the second Gram-positive *S. aureus* was inhibited to a generally lower extent when higher amounts of QQ proteins were added. These findings further emphasize that QQ-2, QQ-6, QQ-8 and QQ-12 significantly interfere with AI-2 modulated biofilm formation of Gram-negative and Gram-positive model organisms. In particular, QQ-2 showed strongest effects on biofilm formation except for *P. aeruginosa*.

**Figure 2:**
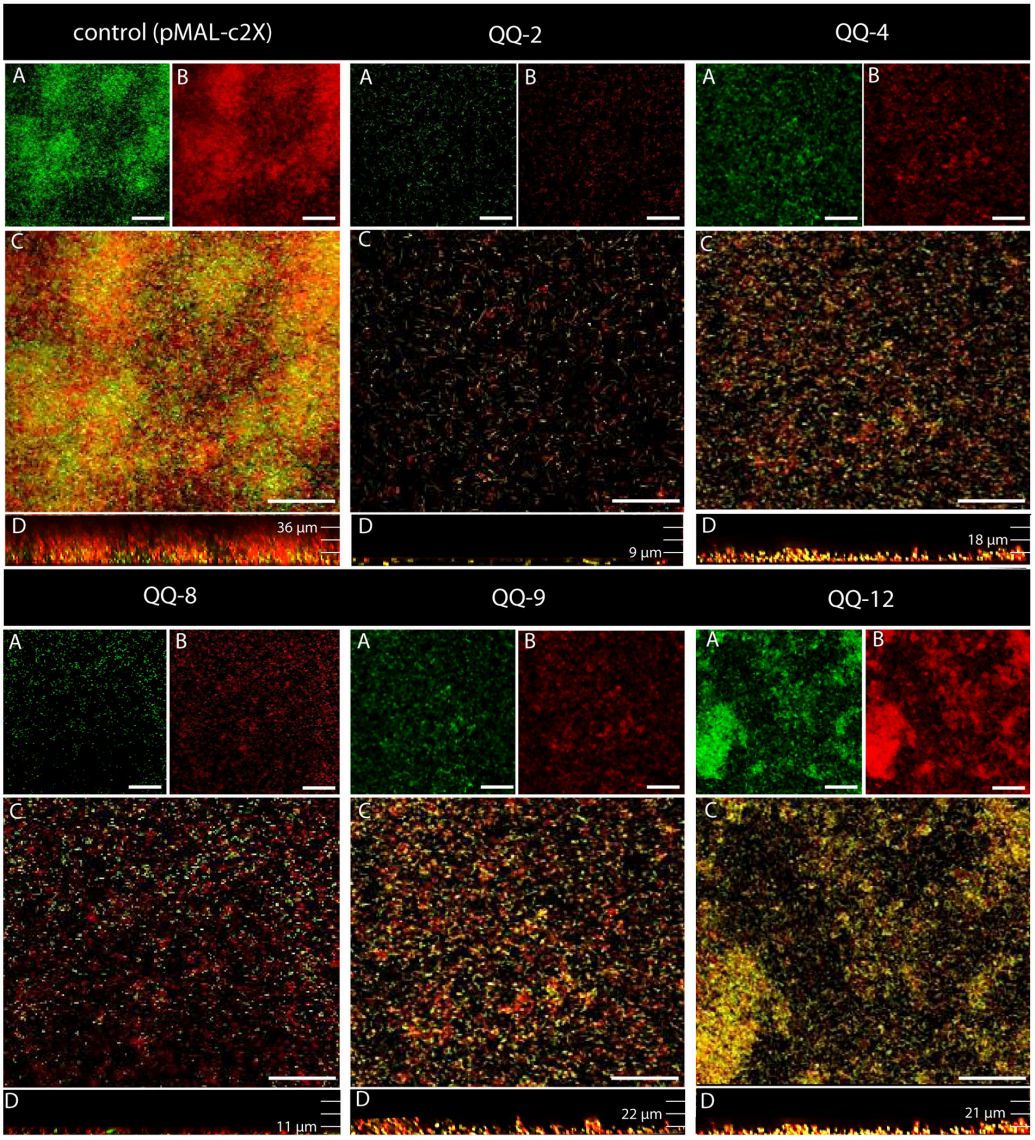
**Biofilm formation of *K. oxytoca* Ma1 expressing QQ-ORFs**. 1.35 × 10^8^ cells of *K. oxytoca* M5a1 expressing selected QQ-ORFs from pMAL-fusion vectors were introduced into the chamber. After 1 h, flow cells were flowed for 72 h at 30°C with 20 mL/h GC medium containing 30 μM IPTG. Biofilms were stained with Live/Dead viability Kit. The 3D biofilm structure was recorded with Leica TCP Confocal Laser Scanning Microscope (Leica) and analyzed with Leica software. CLSM image showing **(A)** live cells stained with Syto9 (green) and **(B)** propidium iodide stained dead cells (red). **(C)** Overlay of images A and B. **(D)** Overlaid side view of the biofilm. Scale bars represent 50 μm.

To further validate and evaluate the effects of the identified QQ proteins on biofilm formation, the respective genes were heterologously expressed from plasmids (pRS611 – pRS622) in *K oxytoca* M5a1, which forms stable biofilms under minimum nutrient conditions modulated by AI-2 QS (Balestrino et al., 2005; Zhu et al., 2011). First, general inhibitory effects on growth caused by the induction of QQ-ORFs from the respective plasmids were excluded by evaluating batch cultures (Figure S2). However, expression of *qq-2, qq-4, qq-6*, and *qq-8* induced to different levels from plasmids (pRS611 – pRS621) significantly inhibited biofilm formation under static conditions in microtiter plates (see Figure S3). To verify these results, continuous flow cell experiments were performed using *K. oxytoca* M5a1 carrying the respective plasmids, followed by biofilm structure analysis after 72 h using Confocal Laser Scanning Microscopy (CLSM). *K. oxytoca* carrying the empty vector pMAL-c2X (control) formed biofilms with an average thickness of 44 ± 4 μm representing a volume of 23 ± 3 μm^3^/μm^2^. In general, the expression of all tested QQ-ORFs had significant influence on thickness (unpaired *t*-test *P*-values < 0.02) as well as on structure of the biofilm (see Table 3, exemplarily shown in Figure 2). Particularly, expression of QQ-2 and QQ-8 reduced biofilm thickness by 75% and the volume up to 86%. Further analysis of the 3D-structures of established biofilms demonstrated that the *K. oxytoca* control formed compact biofilms with a wavy structure. However, due to the expression of QQ-2 and QQ-8 complete inhibition of biofilm formation was revealed. This is shown by the adhesion of only a few living single cells to the glass surface of the flow cell (Figure 2). Biofilms of recombinant *K. oxytoca* expressing QQ-4 – QQ-7 were significantly reduced in thickness and volume (each up to 60 %), although high cell numbers were detected in the xy-plane (including high amounts of dead cells). Expressing QQ-3, QQ-9 and QQ-12 in *K. oxytoca* led to an average biofilm thickness of 22 μm, although only few cells were detected in the xy-plane resulting in a reduction of volume (see Table 3). These findings strongly indicate that all identified ORFs with significant QQ activity indeed affected biofilm formation of *K. oxytoca*, suggesting effective inhibitory effects on biofilms by QQ activities. The different QQ-proteins inhibited to various degrees, and probably at different stages of AI-2 modulated biofilm formation (see CLSM image examples in Figure 2). In addition, expression in *K. oxytoca* might also result in individually different solubility and active fractions of the specific metagenomic proteins.

**Table 3:** **Evaluation of *K. oxytoca* M5a1 biofilm formation in the presence of indigenous expressed QQ-proteins**.

**QQ protein (plasmid)**	**Biofilm thickness [μm]**	***P*-value**	**Volume [μm^3^/ μm^2^]**	***P*-value**	**Biofilm structure**
none (wild type)	41 ± 5	–	22 ± 3	–	Compact 3D-biofilm with wavy structures
MBP (pMAL-c2X)	44 ± 4	–	23 ± 4	–	Compact 3D-biofilm with wavy structures
QQ-2 (pRS611)	10 ± 1	<0.0001	3 ± 1	<0.0001	Reduced cell adhesion
QQ-3 (pRS612)	22 ± 2	<0.0001	16 ± 4	<0.0127	Compact monolayer without wavy structures
QQ-4 (pRS613)	17 ± 2	<0.0001	9 ± 2	<0.0001	Compact monolayer without wavy structures
QQ-5 (pRS614)	19 ± 3	<0.0001	11 ± 3	<0.0002	Compact monolayer without wavy structures
QQ-6 (pRS615)	19 ± 2	<0.0001	12 ± 2	<0.0001	Compact monolayer without wavy structures
QQ-7 (pRS616)	19 ± 2	<0.0001	13 ± 3	<0.0006	Compact monolayer with few but multi-layered cell aggregates
QQ-8 (pRS617)	12 ± 1	<0.0001	6 ± 2	<0.0001	Reduced cell adhesion
QQ-9 (pRS618)	25 ± 3	<0.0001	13 ± 2	<0.0003	Compact monolayer without wavy structures
QQ-12 (pRS621)	22 ± 1	<0.0001	17 ± 3	<0.0148	Several layers of cells with areas without cell adhesion

Flow cells were inoculated with 1.35 × 10^8^ cells of K. oxytoca M5a1 expressing MBP-QQ-ORFs from plasmids. After 1 h, flow cells were flowed for 72 h at 30°C with 20 mL/h GC medium containing 30 μM IPTG. Biofilms were analyzed after 72 h in at least three biological replicates, each with two technical replicates (see Materials and Methods). Average values are depicted with ± standard deviations. Average thickness and volume of MBP control and QQ biofilms are significantly different (p < 0.02, unpaired t-tests).

### Effective biofilm inhibition by surface-immobilized QQ-2

**Figure 3:**
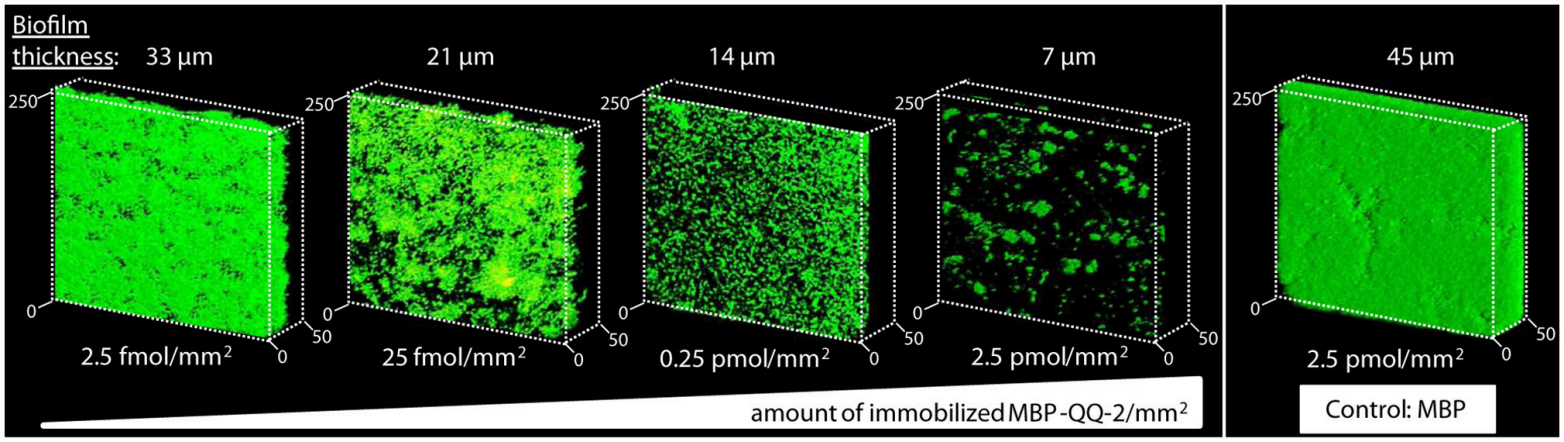
**Effects of immobilized MBP-QQ-2 on biofilm formation of *K. oxytoca***. Purified MBP-QQ-2 was covalently linked to slide surfaces of flow cells using concentrations of 2.5 fmol/mm^2^ – 2.5 pmol/mm^2^. 1.35 × 10^8^ cells of *K. oxytoca* were supplemented and after 1 h, flow cells were flowed for 72 h at 30°C with 20 mL/h GC medium. Biofilms were stained with Syto9. Images were recorded with Leica TCP Confocal Laser Scanning Microscope (Leica) and analyzed with Leica software. 3D CLSM images showing live cells stained with Syto9 (green) were scanned along the biofilm depth (z-axis). Scale μm.

*In vivo* and *in vitro* studies demonstrated that QQ-2 significantly interferes with AI-2 QS most likely due to an oxidoreductase activity. QQ-2 appears to be highly effective regarding its ability to prevent mostly AI-2 modulated biofilm formation of several model organisms. Thus, QQ-2 was further evaluated for biotechnological applications aiming to prevent biofilm formation. First, protein stability of purified MBP-QQ-2 was analyzed using the reporter system (Weiland-Bräuer et al., 2015) which showed that at 30°C up to 72 h 75% of the initial activity and at 4°C up to 4 weeks 80% of the activity was detectable, indicating that the protein is quite stable. Next, purified MBP-QQ-2 was immobilized on the glass cover slips of flow cells by chemically cross-linking the protein to the pretreated surface (layer-by-layer technology, see Materials and Methods). The effects of different amounts of immobilized purified MBP-QQ-2 on the biofilm formation of *K. oxytoca* in the flow cell system in comparison to immobilized control protein (MBP) are depicted in Figure 3. The control treatment with 2.5 pmol/mm^2^ immobilized MBP resulted in compact 3D-biofilms of *K. oxytoca* with 45 μm thickness representing a volume of 21 μm^3^/μm^2^, whereas cell adhesion and formation of micro-colonies was efficiently prevented by equal amounts of immobilized MBP-QQ-2. With decreasing amounts of immobilized QQ-2, the inhibitory effects decreased in a concentration dependent manner resulting in a 33 μm thick and compact 3D-biofilm at 2.5 fmol QQ-2/mm^2^. The finding that the degree of inhibition correlates with the amount of immobilized QQ-2 molecules strongly indicates an enzymatic based inhibition process with a specific enzyme kinetic. Consequently, immobilizing the protein by cross-linking to various abiotic surfaces appears to be a promising approach for biotechnological application.

The effects of immobilized QQ-2 were further evaluated in respect to biofilm prevention of pathogens, i.e., *Klebsiella* strains isolated from patients with urinary tract infections. Flow cell experiments with immobilized MBP-QQ-2 (2.5 pmol/mm^2^) resulted in prevention of AI-2 modulated biofilm formation to varying degrees (summarized in Table 4, depicted in Figure S4). In cases of ESBL-Nos. 134, 149, cell adhesion to the surface was nearly completely prevented; and only monolayers were formed without maturing to a compact 3D-biofilm in cases of ESBL-Nos. 81, 126, 130, 147. For the remaining isolates (ESBL-Nos. 92, 150) only small effects on biofilm formation were revealed, although a reduction of biofilm thickness of at least 50 % and of volume from 27 to 95% were observed. Unpaired *t*-tests revealed high significance (*P*-values < 0.02) for the compared biofilm characteristics. Characteristics of control biofilms formed on immobilized MBP and respective immobilized QQ-2 were included in the statistical tests for all *Klebsiella* strains. Overall, this evaluation demonstrates that QQ-2 has the ability to also inhibit clinically relevant biofilms connected with disease.

**Table 4:** **Effects of immobilized MBP-QQ-2 on clinical *Klebsiella* isolates**.

**Clinical isolate**		**Biofilm parameters in the presence of immobilized QQ-2**			**Biofilm parameters in the presence of immobilized MBP**			**Mean reduction of biofilm parameter (immobil. QQ-2 vs. immobil. MBP)**	
**Species**	**ESBL-No**.	**T [μm]**	**V [μm^3^/μm^2^]**	**Characterization**	**T [μm]**	**V [μm^3^/μm^2^]**	**Characterization**	**T (%)**	**V (%)**
Wildtype *K. oxytoca* M5a1		5 ± 2	1 ± 1	Reduced cell adhesion	45 ± 4	21 ± 2	Compact 3D-biofilm with wavy structure	**89** ± 4	**95** ± 4
*K. pneumonia*	134	8 ± 3	1 ± 1	Reduced cell adhesion	41 ± 3	19 ± 2	Compact 3D-biofilm with wavy structure	**81** ± 6	**95** ± 4
*K. pneumonia*	81	20 ± 4	10 ± 2	Monolayer with few multilayered cell aggregates	44 ± 4	20 ± 2	Compact 3D-biofilm with wavy structure	**55** ± 5	**50** ± 1
*K. pneumonia*	126	11 ± 2	8 ± 2	Monolayer with areas without cell adhesion	48 ± 2	23 ± 1	Compact 3D-biofilm with wavy structure	**77** ± 3	**65** ± 7
*K. oxytoca*	149	7 ± 3	2 ± 1	Reduced cell adhesion	46 ± 3	20 ± 2	Compact 3D-biofilm with wavy structure	**85** ± 5	**90** ± 4
*K. pneumonia*	130	15 ± 3	6 ± 1	Aggregates with multilayered cells	48 ± 4	22 ± 2	Compact 3D-biofilm with wavy structure	**69** ± 4	**73** ± 2
*K. pneumonia*	147	10 ± 4	8 ± 3	Monolayer without a wavy structure	43 ± 4	20 ± 2	Compact 3D-biofilm with wavy structure	**77** ± 7	**60** ± 10
*K. pneumonia*	150	15 ± 4	13 ± 4	Compact monolayer without wavy structure	44 ± 3	21 ± 1	Compact 3D-biofilm with wavy structure	**66** ± 6	**38** ± 15
*K. pneumoniae*	92	24 ± 5	16 ± 2	Several layers of cells with wavy structure	49 ± 5	22 ± 3	Compact 3D-biofilm with wavy structure	**51** ± 5	**27** ± 1

Biofilm formation in flow cells in the presence of 250 pmol/mm^2^ immobilized MBP-QQ-2 was analyzed after 72 h growth in GC medium at 30°C. Biofilm analysis was performed in two independent biological experiments with each two technical replicates. Average values for biofilm thickness (T) and volume (V) are depicted with ± standard deviations. Both parameters are significantly different between biofilms formed on immobilized MBP and respective immobilized QQ-2 (unpaired t-test, p < 0.02). Bold values describe the mean reduction of biofilm thickness (T) and volume (V) in percent. Those values are summarizing the whole information of the table in one column and are thus highlighted by bold notation.

### Molecular and biochemical characterization of QQ-2

The highly attractive candidate QQ-2 was further characterized by detailed molecular and biochemical analysis aiming to gain insight into the underlying QQ mechanism. Nucleotide and aa sequence based classifications assigned QQ-2 to the large superfamily of short-chain reductases (SDR) using NCBI Conserved Domain Search. QQ-2 contains a single domain with a structurally conserved Rossmann fold, an NAD(P) binding region, and a structurally diverse C-terminal region, which is typical for reported SDRs (Jörnvall et al., 1995). SDRs catalyze a wide range of reactions involved in the metabolism of steroids, cofactors, carbohydrates, lipids, aromatic compounds, and amino acids, and act in redox sensing (Hong et al., 2007). QQ-2 comprises highest homologies to oxidoreductases of the fatty acid metabolism catalyzing an NAD(P)-dependent oxidation of a hydroxyl-group to a keto-group (see phylogenetic tree of SDR representatives in Figure S5).

**Figure 4:**
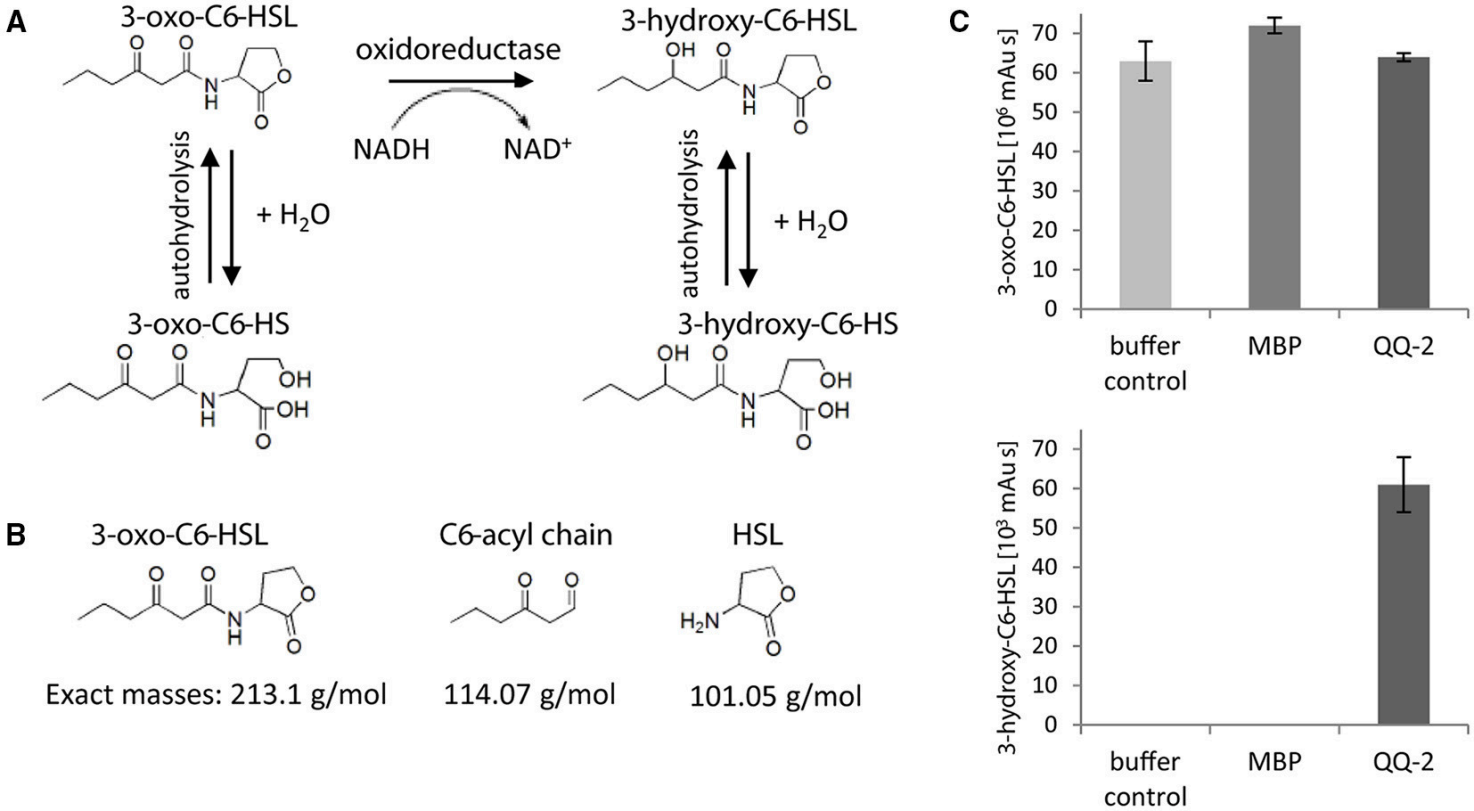
**Oxidoreductase activity of QQ-2. (A)** Predicted autohydrolysis and putative reduction of 3-oxo-C6-HSL by QQ-2. **(B)** Molecules detected by HPLC/MS/MS analysis after incubation of 3-oxo-C6-HSL with QQ-2. Exact masses of substrate and products are depicted. **(C)** AHL degradation assay using 50 μM 3-oxo-C6-HSL without enzyme (buffer control) or 1 mg/mL enzyme (MBP, control; QQ-2, oxidoreductase) in 0.1 M phosphate buffered saline (pH 7.0) at 30°C for 14 h. Peak areas of reactant 3-oxo-C6-HSL (left panel; depicted as 10^6^ mAU × s) and product 3-hydroxy-C6-HSL (right panel; depicted as 10^3^ mAU × s) are depicted. Three biological replicates each with three technical replicates were analyzed.

To detect the assumed oxidoreductase activity of QQ-2, optical test assays were performed using the respective signaling molecules, AHL and AI-2, as substrates and monitoring NADH oxidation by decrease of absorbance at 340 nm (see Materials and Methods). The apparent oxidoreductase activity was calculated with 28.0 ± 1.8 mU/mg using 3-oxo-C6-HSL as substrate, and with 6.0 ± 0.8 mU/mg using 4-hydroxy-5-methyl-3-furanone. These low activities might be either due to non-optimal assay conditions (e.g., substrates not reaching KM concentrations) or indicate that the QQ activity represents a site activity of the protein. In control reactions containing exclusively NADH, or NADH with purified MBP or QQ-2 in the absence of signaling molecules, no change in absorbance was obtained. Ultimately, oxidoreductase activity of QQ-2 was verified by analyzing the reaction products of 3-oxo-C6-HSL in the presence of QQ-2 and NADH by HPLC/MS/MS (see Materials and Methods). Assuming that QQ-2 shows oxidoreductase activity and reduces AHL with NADH as electron donor, one would predict the generation of 3-hydroxy-C6-homoserine lactone (OHHL) as a potential product. In addition, both the substrate AHL and the respective reduced product OHHL can spontaneously hydrolyze to the corresponding homoserines (see Figure 4A). Quantifying AHL amounts by HPLC/MS/MS measurement (see Figure 4B) after the incubation demonstrated that autohydrolysis of AHL occurred independently of QQ-2 and to very similar amounts as the detected AHL decrease in the presence of QQ-2 (see buffer control, MBP in Figure 4C upper panel). Consequently, due to the small differences between autohydrolysis and enzymatic reduction, the apparently small decrease of AHL based on enzymatic reduction was not detectable. However, the reduced product OHHL was exclusively detected in significant amounts when AHL was incubated in the presence of QQ-2 (Figure 4C lower panel), indicating that QQ-2 reduces the signaling molecule AHL to OHHL.

**Figure 5:**
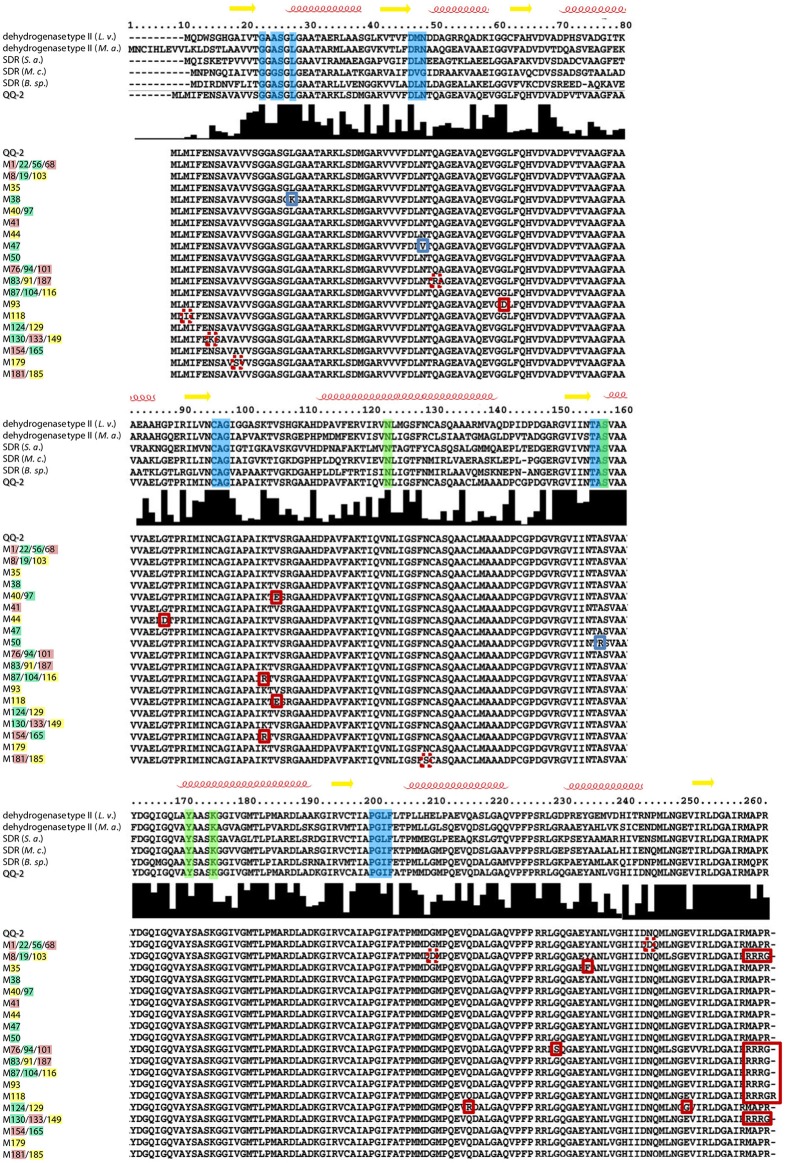
**Alignment of QQ-2 homologs and QQ-2 mutants**. Alignment of QQ-2 with homologs including elements of the secondary structure (α*-helices, red;* β*-sheet, yellow*) is depicted. Predicted NAD(P) binding sites are illustrated with *blue boxes* and predicted active sites with *green boxes*. ClustalX (Larkin et al., 2007) histogram of sequence conservation is shown underneath the alignment. Biological sources and GenBank accession codes for homolog sequences are as follows: dehydrogenase type II, *Loktanella vestfoldensis* (*L. v*.) (ZP_01004376.1); dehydrogenase type II, *Maritimibacter alkaliphilus* (*M. a*.) (ZP_01012736.1); SDR, *Stappia aggregata* (*S. a*.) (ZP_01545205.1); SDR, *Mesorhizobium ciceri* (*M. c*.) (YP_004141279.1); SDR, *Burkholderia* sp. (*B.* sp.) (ZP_03269755.1). In the lower panel, alignment of QQ-2 with corresponding protein sequences of mutagenized clones (M1-M187), which lost AHL- (clone designation highlighted in *green*), AI-2 (*red*) or simultaneous (*yellow*) QQ activity shows positions of mutations highlighted by *boxes.* Based on conservation and mutation frequency (mutant clones harboring identical mutations are separated by slash), mutations in NAD(P) binding sites are displayed with *blue boxes*, whereas mutations most likely resulting in conformational changes of the protein are marked with *red boxes*. Sequence alignments and visualization of secondary structures were performed using STRAP—Interactive Structure based Sequences Alignment Program (http://www.bioinformatics.org/strap/).

**Figure 6:**
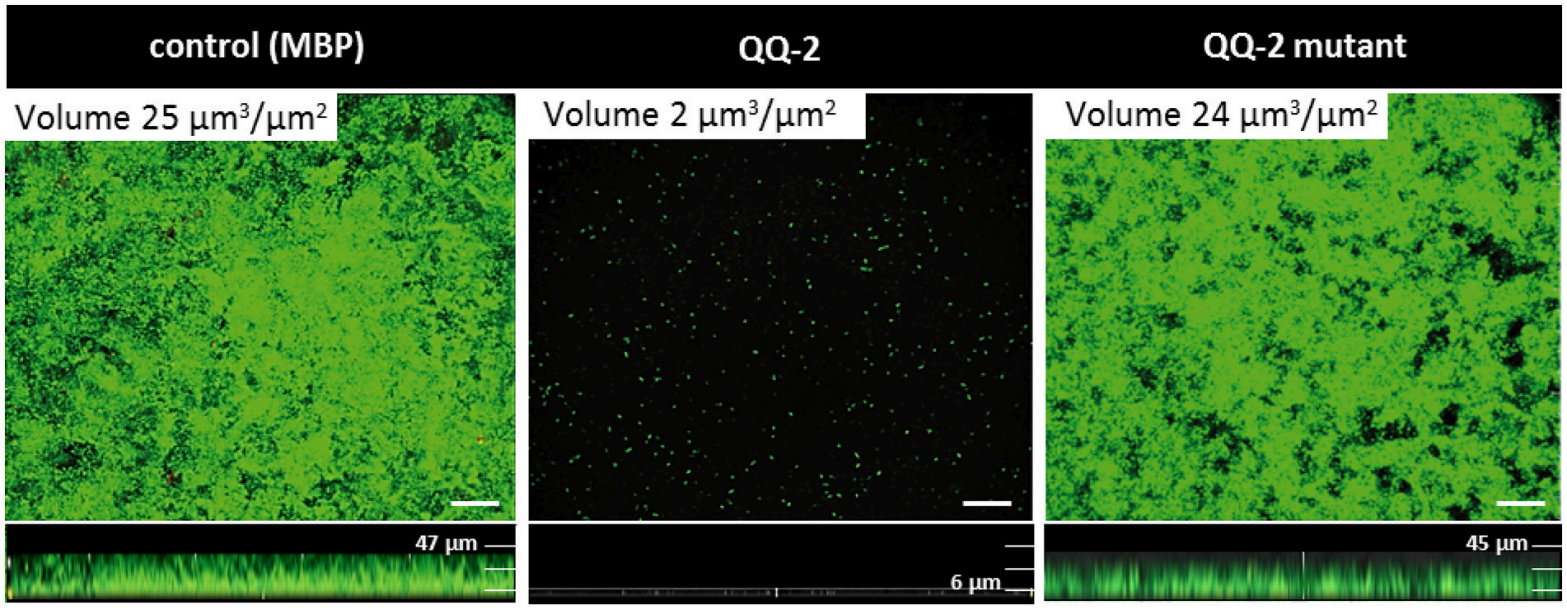
**Immobilized mutated QQ-2 restores biofilm formation of *K. oxytoca* M5a1 in continuous flow cells**. Purified mutated MBP-QQ-2 derivate (N48V), MBP-QQ-2 and MBP were covalently linked to surfaces of flow cells (2.5 pmol/mm^2^). 1.35 × 10^8^ cells were supplemented into the cell and after 1 h flowed with 20 mL/h GC medium. After 72 h biofilms were stained with Live/Dead viability Kit. All images were recorded with Leica TCP Confocal Laser Scanning Microscope (Leica) and analyzed with Leica software. 3D CLSM images showing overlays of Syto9 (green) stained live cells and propidium iodide (red) stained dead cells. Scale bar 50 μm.

Aiming to identify aa of QQ-2 essential for QQ activity, a general mutagenesis was performed using a PCR approach with decreased *Taq* polymerase accuracy (see Materials and Methods), resulting in an overall mutation rate between 1 to 6 point mutations within the QQ-2 ORF (771 nt). The QQ-2 ORF of pRS611 was replaced by the respective mutated PCR fragments and resulting clones were screened for QQ activity. In total, cell extracts of 940 mutant clones resulting from five independent PCR amplifications were analyzed using the reporter strains AI1-QQ.1 and AI2-QQ.1. This led to the identification of 188 clones which showed absolutely no QQ activities. Sequencing of the respective mutated *qq-2* genes identified several essential point mutations. Overall, 14 single nucleotide mutations were identified leading to a complete loss of AHL-QQ activity of the protein, 10 single nucleotide mutations led to the loss of AI-2-QQ activity, whereas 12 mutations were identified leading to simultaneous loss of both QQ activities using the reporter assays. In Figure 5, the respective mutant sequences are illustrated in an alignment compared to the five best homologs of QQ-2. The alignment indicated that the affected aa in QQ-2 which lead to loss of function are either located in the predicted NAD(P) binding sites (*blue boxes*), highly conserved regions or disordered regions often important for protein function such as allosteric regulation and enzyme catalysis (Kamerlin and Warshel, 2011) (see Figure 5 and Figure S6). In addition, flow cell experiments with *K. oxytoca* M5a1 with an immobilized mutated and thus inactive form of QQ-2 (N48V, mutation in NAD(P) binding site) resulted in restored biofilm formation (Figure 6). This experiment clearly demonstrated that biofilm inhibition crucially depends on the QQ activity of the QQ protein. Additionally, an impaired adhesion of *K. oxytoca* cells to the protein surface in comparison to the glass flow cell surface can be excluded.

## Discussion

### First metagenomic-derived QQ enzymes simultaneously interfering with AHL- and AI-2 QS

In general, QQ is considered as a natural strategy evolved either to recycle or degrade own QS signals or to defend competing microorganisms. Numerous QS interfering enzymes and small molecules have been identified since the first report of enzymatic degradation of AHL signals by soil bacterial isolates of the genera *Variovorax* and *Bacillus* (Dong et al., 2000; Leadbetter and Greenberg, 2000). Besides, only very few AI-2 interfering mechanisms have been reported so far in contrast to the various identified AHL-quenching compounds. These AI-2 quenching mechanisms are mainly based on interference with AI-2 synthesis (Shen et al., 2006; Widmer et al., 2007; Skindersoe et al., 2008) or represent antagonistic small molecules (Ganin et al., 2009; Lowery et al., 2009; Vikram et al., 2011; Roy et al., 2013; Yadav et al., 2014). Only a few QQ enzymes degrading or modifying AI-2 are currently reported. Enzymatic AI-2 QQ activities are suggested for native LsrG and LsrK from *E. coli*. LsrG is a cytoplasmic protein potentially involved in the degradation of phospho-AI-2 in *E. coli* (Xavier et al., 2007). LsrK is the enzyme responsible for phosphorylation of AI-2 following the import into the cell (Xavier et al., 2007; Marques et al., 2011). Roy et al. showed that the addition of purified LsrK to *E. coli* or Salmonella Typhimurium cultures inhibits QS activation by blocking AI-2 import (Roy et al., 2010). Supplementing with LsrK also blocks QS under co-culture conditions (Roy et al., 2010), suggesting that LsrK could be useful for quenching the underlying AHL and AI-2 cell–cell communication.

Although providing a unique opportunity to identify naturally developed QQ mechanisms, only a small proportion of identified QQ compounds have been discovered using metagenomic approaches. Screening of soil metagenomes mainly extended the diversity of AHL-lactonases by identifying the metagenomic enzymes QlcA, BpiB01, BpiB04, and BpiB05 (Riaz et al., 2008; Schipper et al., 2009; Bijtenhoorn et al., 2011b). The *qlcA* gene product efficiently quenches QS regulated pathogenic functions when expressed in the plant pathogen *Pectobacterium carotovorum* (Riaz et al., 2008), whereas BpiB01, BpiB04 and BpiB05 have been shown to degrade N-(3-oxooctanoyl)-L-homoserine lactone and thus inhibit motility and biofilm formation of the opportunistic human pathogen *P. aeruginosa* (Schipper et al., 2009; Bijtenhoorn et al., 2011b).

Aiming to establish alternative strategies to prevent unfavorable biofilm formation, several novel metagenomic-derived, non-toxic biomolecules simultaneously interfering with AI-2 and AHL based QS were identified in this study by screening metagenomic large-insert libraries of various environments using recently established reporter systems (Weiland-Bräuer et al., 2015). Overall, 100 QQ-active metagenomic clones out of 46,400 were identified confirming the assumption that metagenomes are a rich source for identifying novel naturally occurring QQ compounds with high rates. It also demonstrates that using metagenomics to identify novel QQ biomolecules is a promising approach. Detailed analysis of a selected number of attractive QQ-ORFs ultimately led to the identification of nine QQ proteins comprising the ability to significantly inhibit biofilm formation of several clinically relevant model organisms most likely due to interference with AI-2 and AHL based QS. On the one hand, based on sequence-based prediction these novel QQ proteins enormously extended the diversity of QQ enzymes (e.g., oxidoreductases, amidases, aminotransferases, and proteases). On the other hand, besides AHL interfering activities we identified proteins interfering with the universal AI-2 signaling molecule, which is to our knowledge the first description of metagenomic derived proteins conferring AI-2 QQ activity. Moreover, even simultaneous AHL- and AI-2 interfering metagenomic QQ-proteins were identified.

The results from the initial reporter screen perfectly match for all analyzed QQ proteins with the subsequently performed biofilm assays, except for QQ-3 and QQ-8. The reporter assay demonstrated exclusive QQ activity against AI-2 for QQ-3 and against AHL for QQ-8. QQ-3 showed just slight effects on all biofilm forming models, thus we concluded that this protein is not a potent QQ protein for use in biotechnology. QQ-8 showed drastic biofilm inhibition of all model systems except for P. aeruginosa. This unexpected finding that biofilm inhibition occurred when its formation is based on AI-2 or AI-2/peptide QS might be due to the fact that additional side/secondary activities of the protein might be only detectable under the conditions used in the biofilm assays using high amounts of purified proteins. Besides, QQ-8 was predicted as protease and thus might also affect biofilm formation of Gram-positives by interfering with signaling peptides, an activity which is generally not picked up in the initial reporter screen.

The identified QQ-2 demonstrated to simultaneously interfere with AHL- and AI-2 showed by far the highest inhibitory effect on AI-2 modulated biofilm formation (Figures 1, 2). Moreover, we demonstrated that QQ-2 significantly affects biofilm formation of clinical *K. pneumoniae* isolates derived from patients with urinary tract infections (Table 4 and Figure S4). This is notable, since *Klebsiella* strains are often resistant to first-line antibiotics due to expression of extended spectrum beta-lactamase enzymes (ESBLs) and the development of resistance to carbapenems.

### Oxidoreductase QQ-2 efficiently interferes with AI-2 modulated biofilm formation

Reduced biofilm formation as a consequence of enzymatic modification or degradation of signaling molecules is almost exclusively known for AHL (Grandclâment et al., 2016). For instance, it was demonstrated that several lactonases, acylases, and oxidoreductases are able to affect biofilm formation (Fetzner, 2015). The first reported lactonase AiiA from *Bacillus spec.* inhibits biofilm formation of *Vibrio cholera*, the causative agent of water-borne diarrheal disease (Augustine et al., 2010). In the plant pathogen *Erwinia carotovora*, the expression of the AHL-lactonase significantly reduces its virulence by degrading the synthesized AHL-autoinducers (Dong et al., 2000, 2001; Reimmann et al., 2002). *P. aeruginosa* biofilms were inhibited on medical plastic devices by an immobilized porcine kidney acylase (Kisch et al., 2014). To date, several AHL-oxidoreductases are known from various bacteria (Liu et al., 2005; Uroz et al., 2005; Bijtenhoorn et al., 2011b; Hong et al., 2012) modifying the 3-oxo group of the AHL signal with the corresponding substitution to generate the respective 3-hydroxy derivative. The first crystal structure of a QQ oxidoreductase, BpiB09 belonging to the SDR family and inhibiting AHL-mediated biofilm formation of *P. aeruginosa*, has been published (Bijtenhoorn et al., 2011a,b). In addition to enzymatic interference, the synthesis and activity of several non-natural AHLs was reported, significantly reducing biofilm formation for instance of *P. aeruginosa* PA01 (Geske et al., 2005). Halogenated furanones produced by the marine red alga *Delisea pulchra* are capable to inhibit both, AHL and AI-2 QS (Rasmussen et al., 2000; Ren et al., 2001), and they affect growth of Gram-positive bacteria (Mhatre et al., 2014). Recently, Bentley and coworkers have described novel synthetic AI-2 analogs that were capable of inhibiting maturation of *E. coli* biofilms *in vitro* and when combined with antibiotics near minimum inhibitory concentrations, almost completely cleared pre-formed *E. coli* biofilms in a microfluidic device (Roy et al., 2013). By screening a large number of samples from plants, ursolic acid and 7-hydroxyindole were found as inhibitors for enterohemorrhagic *E. coli* biofilm formation by blocking the AI-2 pathway (Ren et al., 2005; Lee et al., 2007). Those examples demonstrate that over the last 15 years, a range of QQ enzymes and inhibitors have been identified from different sources, including both prokaryotic and eukaryotic organisms mainly interfering with the AHL-QS system of Gram-negative bacteria (Hentzer and Givskov, 2003; Zhang, 2003; Zhang and Dong, 2004; Kalia, 2015) which provided a scaffold for many potential biofilm inhibitors (Hentzer et al., 2003; Geske et al., 2008; Romero et al., 2012).

QQ-2 represents the first metagenomic QQ protein affecting AI-2 signaling and AI-2 QS-mediated biofilm formation, demonstrated in this study by using several *in vitro* and *in vivo* assays. The fact that QQ-2 is still active after immobilization over a time period of 72 h and significantly affects AI-2 modulated biofilm formation of clinical Klebsiella isolates, demonstrates its enormous potential for biotechnological application. Overall, we obtained strong evidence that immobilization of QS interfering molecules is a valuable novel approach to reduce bacterial attachment and biofilm formation. Further, an immobilized enzyme like QQ-2 most likely interferes very early with biofilm formation by quenching the involved QS signaling molecules and thus prevents initial adhesion of cells to the surface. Moreover, degradation of signal molecules by immobilized enzymes might still be effective even when the substratum is e.g., covered with cells, as signal molecules are able to diffuse toward the enzymes.

Additionally, QQ-2 was assigned as oxidoreductase due to displayed sequence similarity and classification to the NADB-Rossmann superfamily and to 3-ketoacyl-(acyl-carrier-protein) reductases using the NCBI Conserved Domain Search (Figure S5). Three further lines of evidence comprising an optical oxidoreductase assay, random mutagenesis and HPLC/MS/MS analysis of reaction products demonstrated that QQ-2 is a novel SDR, identified to reduce AHL and most likely also AI-2. Although the detected QQ-2-dependent AHL degradation rate is low, HPLC/MS/MS demonstrated that the hydroxy-derivative is only generated in presence of active QQ-2. However, reduction or modification of AI-2 by QQ-2 could, not be analyzed with HPLC/MS/MS due to the lack of appropriate analytical methods. Besides, there are just a few methods described for AI-2 detection, e.g., the *V. harveyi in vivo* bioassay (Turovskiy and Chikindas, 2006) and LC-MS/MS in conjunction with selected reaction monitoring (SRM) (Bajad et al., 2006; Eisenhauer, 2011), but these methods could not successfully be applied to detect AI-2 as well as its modification- or reduction-products due to QQ-2 activities in a quantitative manner. This might be due to the fact that in solution AI-2 exists as several stereoisomers in stable equilibria, which makes it difficult to detect and quantify a decrease of a specific AI-2 isomer (Campagna et al., 2009). However, similar to the demonstrated NAD(P)H-dependent AHL reduction by QQ-2, we hypothesize that QQ-2 reduces 4-hydroxy-2,3-pentanedione-5-phosphate (P-DPD, C5-phosphorylated derivative of the open AI-2 form) to 3,4,4-trihydroxy-2-pentanone-5-phosphate, an QS-inactive AI-2 derivative.

## Perspective and conclusion

The development of antibacterial and anti-disease strategies is largely driven by the urgent need of alternative or complementary approaches to presently often ineffective antibiotic treatments. QQ-based strategies, e.g., application or immobilization of QQ, might become an effective alternative strategy to combat bacterial biofilms. In particular, AI-2 mediated QS systems may be important targets for the development of new therapies to control multi-species biofilms. In general, the increasing number of QS interfering enzymes and compounds by far outnumbers those that have been tested for *in vivo* efficiency targeting biofilm formation, demonstrating that more application studies are required. Those studies will allow developing successful, innovative and applicable approaches for biofilm inhibition as an alternative to antibiotics in the future.

## Author contributions

RS and NW conceived the experiments. NW performed all experiments with technical support of NP, except immobilization of proteins and HPLC/MS/MS analyses performed by MK and AL. NW and RS wrote the manuscript.

## Funding

The project was financially supported by *Bundesministerium für Forschung und Bildung* within the *GenoMik Transfer Netzwerk* (ChemBiofilm, support code 0315587B).

### Conflict of interest statement

The authors declare that the research was conducted in the absence of any commercial or financial relationships that could be construed as a potential conflict of interest.
