# Supplementary material for: Highly Effective Inhibition of Biofilm Formation by the First Metagenome-Derived AI-2 Quenching Enzyme
Source: Front Microbiol. 2016 Jul 13;7:1098. doi: 10.3389/fmicb.2016.01098 (PMC4942472; doi:10.3389/fmicb.2016.01098)
Supplement: Supplementary file 1 [file Table1.PDF]

Tab. S1: Characterization of metagenomic libraries and respective identified clones conferring quorum quenching (QQ) activity. The metagenomic large insert libraries were screened for AHL- and AI-2 QQ compounds present in cell extracts and culture supernatants of metagenomic clones. The number of respective active pools of 96 clones and identified single clones are summarized. Positive microtiter plates and clones were confirmed with two additional independent replicates; -, no QQ activity detected.

**Metagenomic library**	**Habitat**	**No. of clones**	**AHL-quenching activity**				**AI-2-quenching activity**			
			active cell extract		active supernatant		active cell extract		active supernatant	
			96er pools	No. of verified individual clones	96er pools	No. of verified individual clones	96er pools	No. of verified individual clones	96er pools	No. of verified individual clones
III	Microbial mat, Black Sea, 230 m depth, 2004	5.856	10	-	4	11	3	-	2	1
IV	Salt Marsh, Hamburger Hallig, Germany, 2005	2.976	3	-	1	-	3	-	11	9
X	*Aurelia aurita* surface, 2006	3.168	3	-	-	-	-	-	-	-
XIII	Cryoconite, 2006	1.152	2	1	2	3	1	-	2	-
XIV	Biofilm of a washing machine	14.784	14	47	8	48	-	-	-	-
XVII	Water column: Stein, Baltic Sea, Germany, 5 m depth, 2008	9.216	16	12	12	4	-	-	-	-
XIX	*Aurelia aurita* surface, 2008	9.216	9	3	17	13	1	3	-	-
