# Supplementary material for: Highly Effective Inhibition of Biofilm Formation by the First Metagenome-Derived AI-2 Quenching Enzyme
Source: Front Microbiol. 2016 Jul 13;7:1098. doi: 10.3389/fmicb.2016.01098 (PMC4942472; doi:10.3389/fmicb.2016.01098)
Supplement: Supplementary file 2 [file Table2.PDF]

Tab. S2: Primer used for cloning QQ-ORFs in pMAL-c2X.

**Primer designation**	**Primer sequence (5´→3´)** (additional restriction recognition sites are underlined)	**pMAL-c2X fusion vector**
IV 5/G8 QQ-2$_{\text{for}\textit{Xmn}\text{I}}$	GAATCGCTTCAATGCTTATGATATTTGAAAA	pRS611
IV 5/G8 QQ-2$_{\text{rev}\textit{Eco}\text{RI}}$	GAATTCTTACCGCGGCGCCATA	
IV5/G7 QQ-3$_{\text{for}\textit{Xmn}\text{I}}$	GAATCGCTTCAATGCACGGTAATGATACCGA	pRS612
IV5/G7 QQ-3$_{\text{rev}\textit{Eco}\text{RI}}$	GAATTCTTATTGTTTTTCTTCTTGTGT	
IV 5/E10 QQ-4$_{\text{for}\textit{Xmn}\text{I}}$	GAATCGCTTCAATGATTCTCACCCATATG	pRS613
IV 5/E10 QQ-4$_{\text{rev}\textit{Eco}\text{RI}}$	GAATTCCTAAATGTCCAGGGTCAGC	
IV 13/B4 QQ-5$_{\text{for}\textit{XmnI}}$	GAATCGCTTCAATGTCCATCAATCACGTT	pRS614
IV 13/B4 QQ-5$_{\text{rev}\textit{Bam}\text{HI}}$	GGATCCTTATTCGGCGGCAGGTTG	
IV 13/B4 QQ-6$_{\text{for}\textit{Xmn}\text{I}}$	GAATCGCTTCAATGTCCATCAATCACGT	pRS615
IV 13/B4 QQ-6$_{\text{rev}\textit{Bam}\text{HI}}$	GGATCCTTATTCGGCGGCAGG	
IV 13/B4 QQ-7$_{\text{for}\textit{Xmn}\text{I}}$	GAATCGCTTCAATGACGCAGTTGTTAAAAG	pRS616
IV 13/B4 QQ-7$_{\text{rev}\textit{Eco}\text{RI}}$	GAATTCTTAGTCCTTCAGTCCCA	
IV 13/B4 QQ-8$_{\text{for}\textit{Xmn}\text{I}}$	GAATCGCTTCAATGGGCAGGGACTTCCG	pRS617
IV 13/B4 QQ-8$_{\text{rev}\textit{Eco}\text{RI}}$	GAATTCCTA ATGTGCAGTCGCAC	
IV 13/B4 QQ-9$_{\text{for}\textit{Xmn}\text{I}}$	GAATCGCTTCAATGTCCGCTACAGTTG	pRS618
IV 13/B4 QQ-9$_{\text{rev}\textit{Eco}\text{RI}}$	GAATTCTCATTGACGTGTCCGT	
IV 13/B4 QQ-10$_{\text{for}\textit{Xmn}\text{I}}$	GAATCGCTTCAATGGGTTTGGTCTTGAACT	pRS619
IV 13/B4 QQ-10$_{\text{rev}\textit{Eco}\text{RI}}$	GAATTCTTA GCC CGC ACG CGC GG	
III 6/G5 QQ-11$_{\text{for}\textit{Xmn}\text{I}}$	GAATCGCTTCAATGCCGGGTTCCAAAAC	pRS620
III 6/G5 QQ-11$_{\text{rev}\textit{Eco}\text{RI}}$	GAATTCTCACATATGCTGTATCCAG	
III 6/G5 QQ-12$_{\text{for}\textit{Eco}\text{RI}}$	GAATTCATGAGACACCATGAGGATT	pRS621
III 6/G5 QQ-12$_{\text{rev}\textit{Bam}\text{HI}}$	GGATCCTCATCTCCCCCTCGACTC	
