# Supplementary material for: Highly Effective Inhibition of Biofilm Formation by the First Metagenome-Derived AI-2 Quenching Enzyme
Source: Front Microbiol. 2016 Jul 13;7:1098. doi: 10.3389/fmicb.2016.01098 (PMC4942472; doi:10.3389/fmicb.2016.01098)
Supplement: Supplementary file 3 [file Table3.PDF]

Tab. S3: HPLC/MS/MS solvent program for AHL and AHS measurements using Agilent 1200 HPLC system with API 2000 triple quadrupole (Agilent Technologies, Böblingen, Germany).

**Time [min]**	**Flow rate [µL/min]**	**Solvent A for AHL measurement [%]**	**Solvent B for AHL measurement [%]**	**Solvent A for AHS measurement [%]**	**Solvent B for AHS measurement [%]**
3	150	50	50	55	45
15	150	50	50	55	45
30	200	5	95	5	95
35	200	5	95	5	95
38	200	50	50	55	45
43	150	50	50	55	45
