# Supplementary material for: Highly Effective Inhibition of Biofilm Formation by the First Metagenome-Derived AI-2 Quenching Enzyme
Source: Front Microbiol. 2016 Jul 13;7:1098. doi: 10.3389/fmicb.2016.01098 (PMC4942472; doi:10.3389/fmicb.2016.01098)
Supplement: Supplementary file 4 [file Table4.PDF]

Tab. S4: HPLC/MS/MS quadrupole settings for AHL and AHS measurements with API 2000 triple quadrupole detector from ABSciex (ABSciex,Framingham, MA, USA). Q1, mass settings quadrupole 1; Q3, mass settings quadrupole 3; DP, declustering potential; FP, focusing potential; EP, entrance potential; CEP, collision cell entrance potential; CE, collision energy; CXP, collision cell exit potentials.

**AHL**	**Q1 [Da]**	**Q3 [Da]**	**DP [V]**	**FP [V]**	**EP [V]**	**CEP [V]**	**CE [V]**	**CXP [V]**
3-oxo-C6-HSL	214.130	102.0	16	310	12	10	17	0
3-oxo-C6-HSL	214.130	113.0	16	310	12	10	23	4
**AHS**	**Q1 [Da]**	**Q3 [Da]**	**DP [V]**	**FP [V]**	**EP [V]**	**CEP [V]**	**CE [V]**	**CXP [V]**
3-oxo-HS	232.131	128.9	16	340	10	14	15	2
3-oxo-HS	232.131	113.1	16	340	10	14	19	2
