# Supplementary figures and images for: Highly Effective Inhibition of Biofilm Formation by the First Metagenome-Derived AI-2 Quenching Enzyme
Source: Front Microbiol. 2016 Jul 13;7:1098. doi: 10.3389/fmicb.2016.01098 (PMC4942472; doi:10.3389/fmicb.2016.01098)

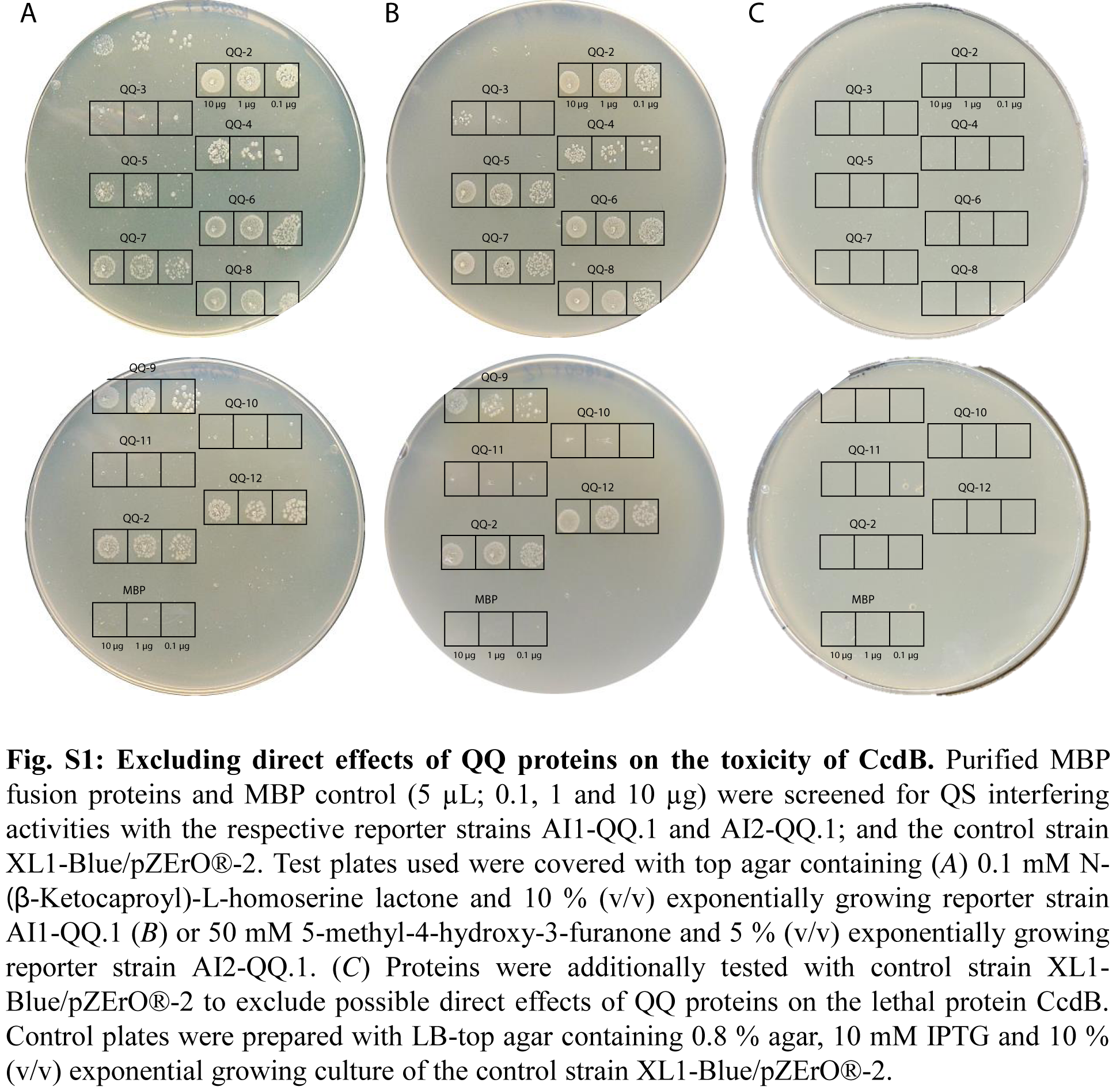

Supplement: Supplementary file 5 [file Image1.TIF]

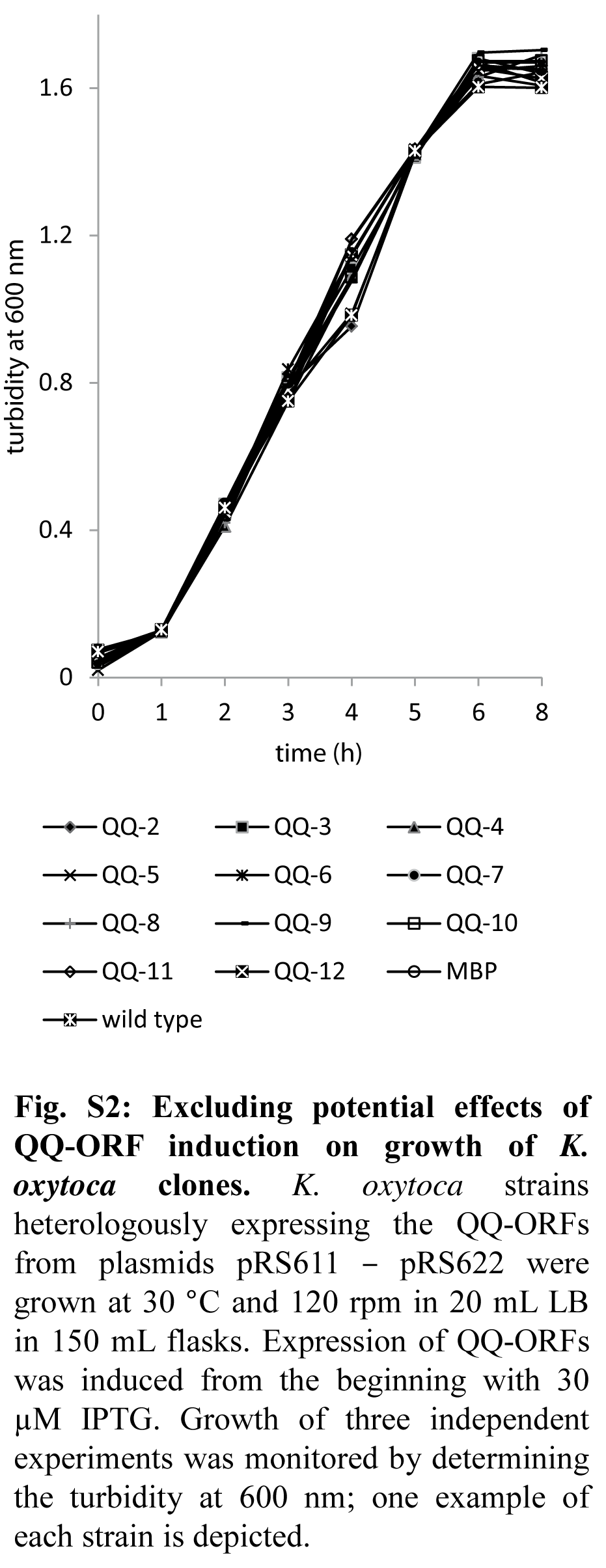

Supplement: Supplementary file 6 [file Image2.TIF]

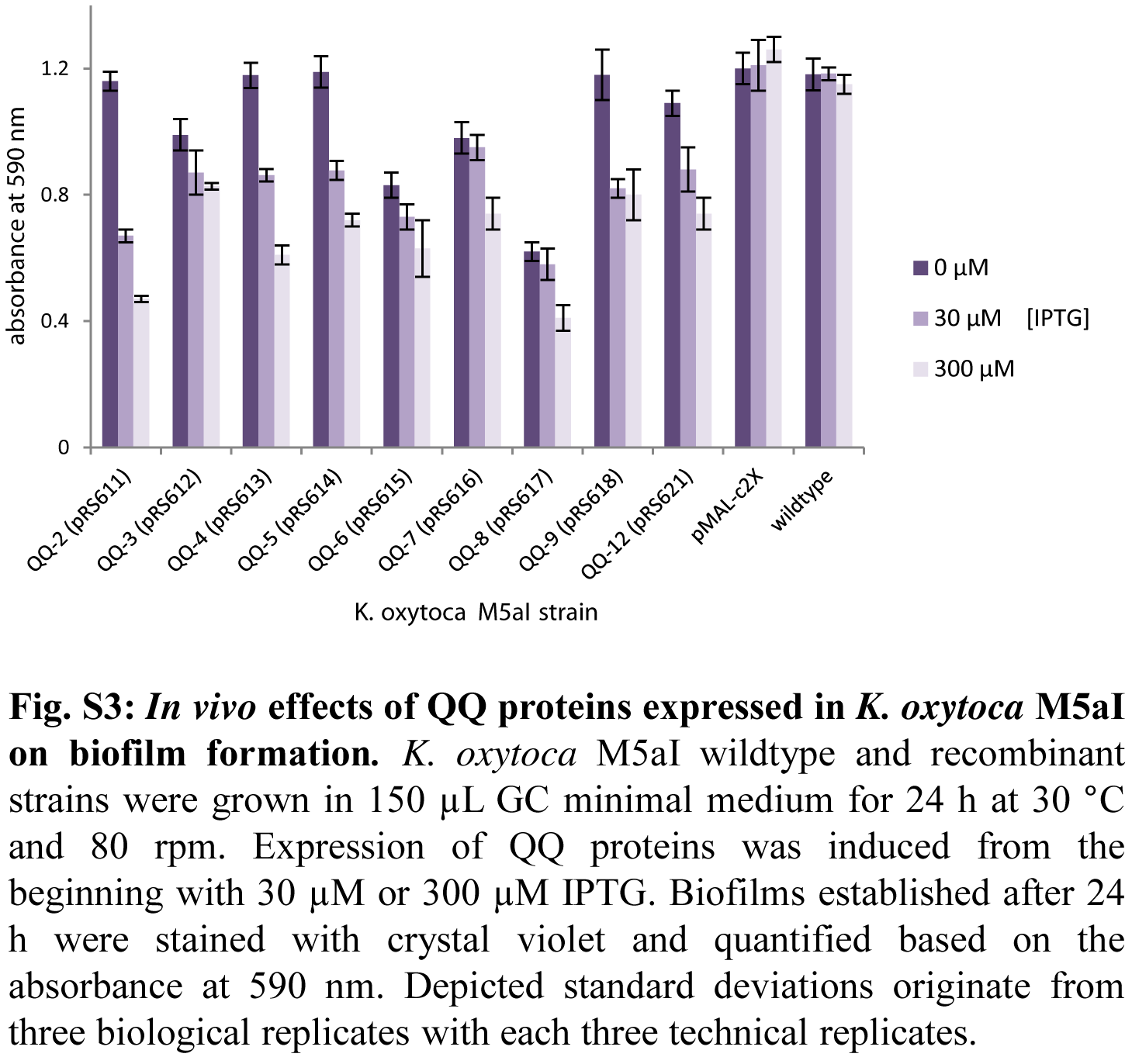

Supplement: Supplementary file 7 [file Image3.TIF]

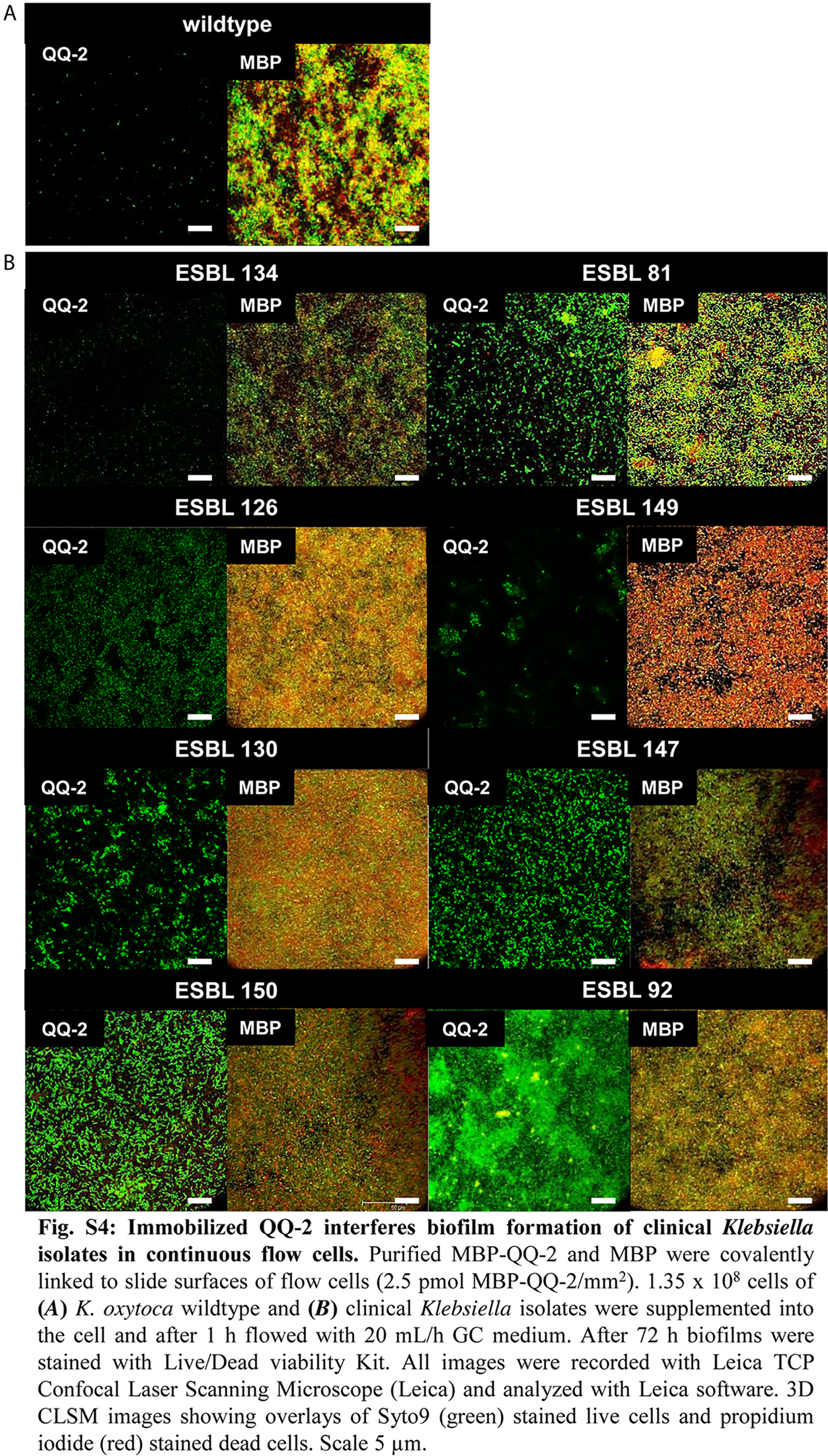

Supplement: Supplementary file 8 [file Image4.TIF]

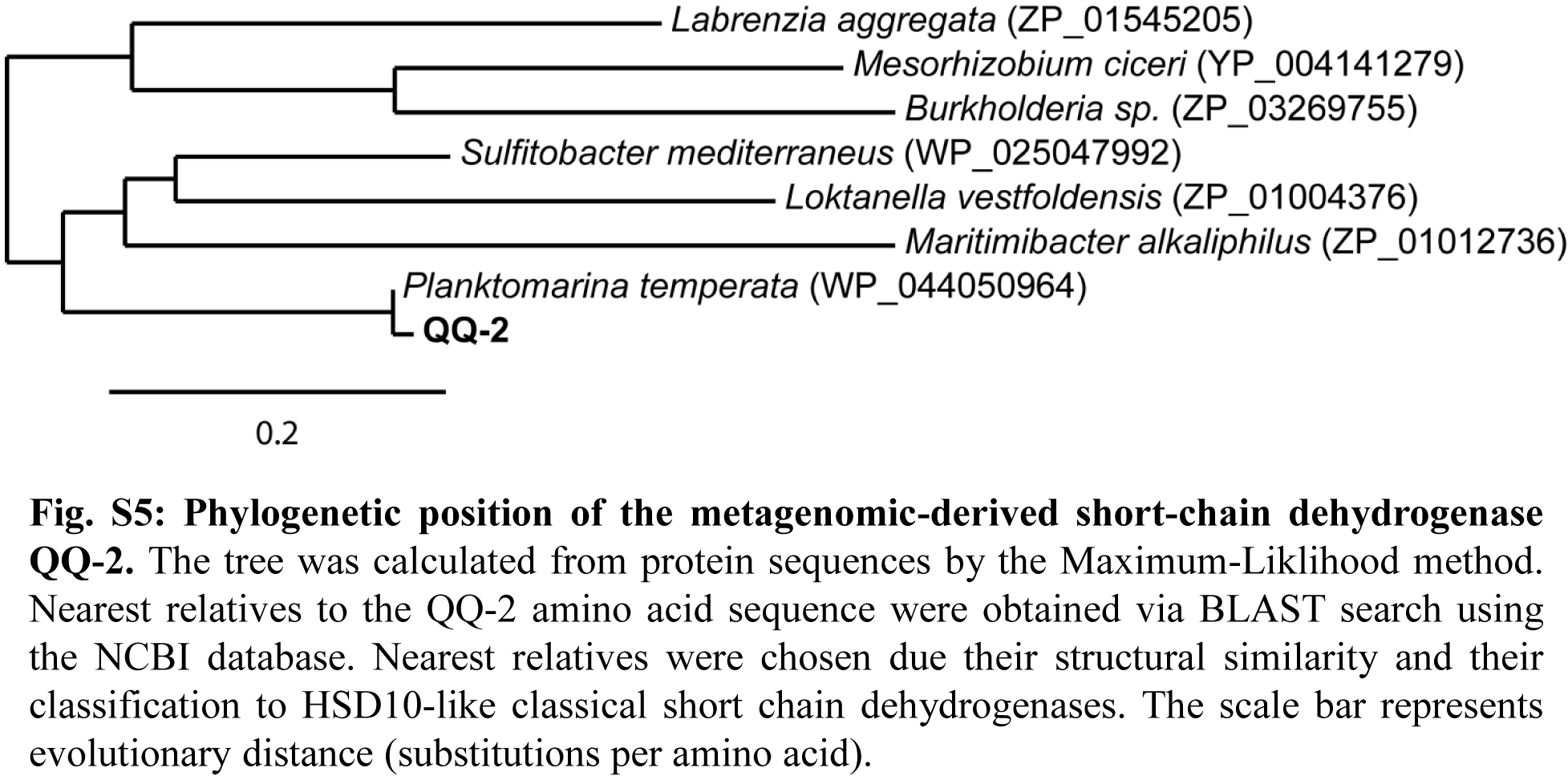

Supplement: Supplementary file 9 [file Image5.TIF]

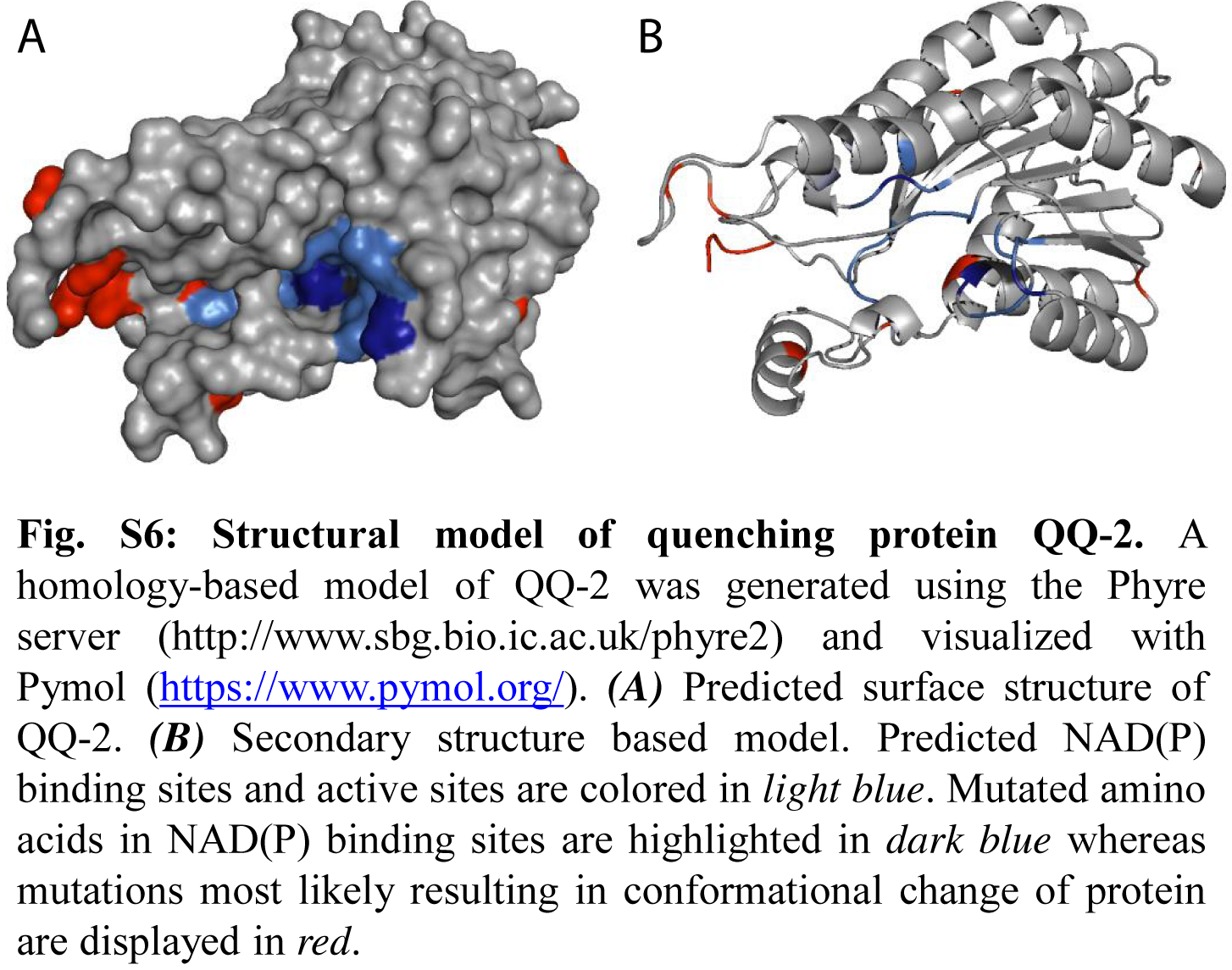

Supplement: Supplementary file 10 [file Image6.TIF]
